# Identification of a Novel Axon Regeneration Role for Noncanonical Wnt Signaling in the Adult Retina after Injury

**DOI:** 10.1523/ENEURO.0182-22.2022

**Published:** 2022-08-10

**Authors:** Ganeswara Rao Musada, Tal Carmy-Bennun, Abigail S. Hackam

**Affiliations:** Bascom Palmer Eye Institute, University of Miami Miller School of Medicine, Miami, FL 33136

**Keywords:** axonal regeneration, noncanonical Wnt signaling, optic nerve crush, retinal ganglion cells, Wnt5a

## Abstract

Canonical and noncanonical Wnt signaling pathways are essential for development and maintenance of the CNS. Whereas the roles of canonical Wnt pathways in neuronal survival and axonal regeneration in adult CNS have been described, the functions of noncanonical Wnt pathways are not well understood. Furthermore, the role of noncanonical Wnt ligands in the adult retina has not been investigated. Noncanonical Wnt signaling shares receptors with canonical Wnt ligands but functions through calcium and c-Jun N-terminal kinase (JNK) signaling pathways. Noncanonical ligands, such as the prototypic ligand Wnt5a, have varying effects in the developing CNS, including inhibiting or promoting axonal growth. To identify a role for noncanonical Wnt signaling in the developed retina after injury, we characterized the effect of Wnt5a on neurite outgrowth in cultured retinal ganglion cell (RGC) neurons and on axonal regeneration in the injured optic nerve in the mouse. Endogenous Wnt5a was upregulated after injury and exogenous Wnt5a significantly enhanced neurite growth of primary RGCs and led to extensive axonal regeneration after optic nerve crush (ONC) injury. Wnt5a also significantly increased RGC survival. Furthermore, Wnt5a induced phosphorylation of CamKII and JNK and induced expression of their downstream pathway components. Therefore, these results demonstrate for the first time that Wnt5a promotes axonal growth and protects RGCs in the adult retina.

## Significance Statement

Adult retinal ganglion cells (RGCs) have poor regenerative abilities after axonal injury. Molecules and signaling mechanisms that induce RGC survival, RGC axon growth and optic nerve regeneration have potential to treat optic neuropathies. In the current study, we identified the neurodevelopment factor Wnt5a as a novel regulator of RGC neurite outgrowth in primary RGC cultures and showed that it induces RGC survival and optic nerve regeneration in an adult optic nerve crush (ONC) mouse model. Furthermore, we demonstrated that Wnt5a activates multiple prosurvival and proregenerative pathways in RGCs and retina. Therefore, these results demonstrate for the first time that Wnt5a, a noncanonical Wnt ligand critical for CNS development, also promotes axonal growth and protects RGCs in the adult retina.

## Introduction

Wnt signaling pathways are highly conserved signaling cascades in the developing CNS that regulate cellular proliferation, differentiation and homeostasis as well as axon extension, growth cone guidance and synaptogenesis (Xiao et al., 2017; Garcia et al., 2018). Wnt pathways are categorized into canonical and noncanonical Wnt signaling based on their dependence or independence, respectively, on levels of the central effector β-catenin. In the canonical Wnt signaling pathway, the interaction of a Wnt ligand such as Wnt3a to one of 10 Frizzled (Fzd) receptors and a Lrp5/6 co-receptor leads to β-catenin stabilization and translocation to the nucleus where it enhances TCF/LCF-mediated transcription (Xiao et al., 2017; Garcia et al., 2018). Canonical Wnt signaling is an essential signaling pathway in the retina and CNS during development and adult tissue homeostasis. Previous studies reported that canonical Wnt signaling induces axon regeneration of the adult optic nerve (Patel et al., 2017) and spinal cord (Gao et al., 2019) and protects neurons through activation of STAT3 and other survival pathways (Moore and Goldberg, 2011; Patel et al., 2017).

The roles of noncanonical Wnt signaling have been defined in the developing CNS and include promoting neurogenesis, tumorigenesis, cellular survival, axonal growth and axon guidance (Bodmer et al., 2009; Li et al., 2009; Blakely et al., 2011; Hutchins et al., 2011; Rosso and Inestrosa, 2013; Arenas, 2014; Kumawat and Gosens, 2016). Noncanonical Wnt ligands mediate both axonal attraction and repulsion depending on expression of certain receptors and signaling components, for example, repelling ventral nerve axons and promoting retinal axon growth across the optic chiasm midline (Bovolenta et al., 2006; Morenilla-Palao et al., 2020). Noncanonical Wnt signaling pathways are mainly categorized into Wnt/planar cell polarity (PCP) and Wnt/Ca^+2^ pathways. The Wnt/PCP pathway is activated after interaction of a noncanonical Wnt ligand such as Wnt5a with a Fzd receptor, leading to activation of a cascade involving Rac family small GTPase 1 (RAC1), the Ras homolog gene family member A (RHOA) and c-Jun N-terminal kinase (JNK). Wnt/Ca^+2^ signaling is activated when Wnt ligands bind to Fzd receptors, Ryk or Ror 1 or 2 receptors, which activates heterotrimeric G-proteins and leads to phospholipase C activation and release of calcium from intracellular stores. CamKII is activated by increased intracellular calcium, and it then activates CREB and NFAT transcription factors, whereas JNK activates the transcription factors cJUN and STAT3 (Moore and Goldberg, 2011; Guo et al., 2021).

The prototypic noncanonical Wnt ligand Wnt5a is expressed in developing retina and brain where it is involved in dopaminergic, sympathetic and cortical neuron survival, axon growth and guidance (Bodmer et al., 2009; Li et al., 2009; Blakely et al., 2011; Hutchins et al., 2011). In the developing retina, Wnt5a regulates retinal ganglion cell (RGC) axon growth and controls appropriate axon pathfinding at the optic chiasm (Morenilla-Palao et al., 2020), and Wnt5a is secreted from rod bipolar cells and regulates proper synapse positioning in the outer plexiform layer (Sarin et al., 2018). In developing and postnatal CNS neurons, Wnt5a exerts its effects through activation of CamKII, PKC, and JNK signaling pathways, which have independently been linked to neuronal survival and axonal growth (Bodmer et al., 2009; Farías et al., 2009; Li et al., 2009; Hutchins et al., 2011). Therefore, Wnt5a and other noncanonical Wnt ligands are upstream of multiple pathways that are critical to neuronal survival and function. However, the role of noncanonical Wnt in neuronal survival and axonal regeneration after an injury in adult CNS has not been studied.

In the present study, we investigated the role of Wnt5a in the adult mouse retina after optic nerve injury and characterized potential underlying molecular mechanisms. We hypothesized that stimulation of noncanonical Wnt signaling in the retina using exogenous Wnt5a would protect RGCs and promote optic nerve regeneration after traumatic optic neuropathy. Our results demonstrated that exogenous Wnt5a induced regenerative pathways including JNK/STAT3 and CamKII/CREB signaling in the retina and significantly increased RGC survival, RGC neurite growth and optic nerve regeneration. Therefore, this study identifies the noncanonical Wnt ligand Wnt5a, previously established as critical to embryonic development, as a novel regulator of neuronal survival and optic nerve regeneration in the adult retina.

## Materials and Methods

### Animals

Procedures performed with mice complied with the Association for Research in Vision and Ophthalmology (ARVO) statement for use of animals in ophthalmic and vision research. The Animal Care and Use Committee at the University of Miami approved all experimental protocols. C57BL/6J mice pups (The Jackson Laboratory, stock #000664) were used to isolate primary RGCs. The same strain of mice was also used for optic nerve injuries and intravitreal injections. Mice were housed in a light and dark cyclic environment (12/12 h light/dark) with *ad libitum* access to food and water. Male and female mice were used in all experiments, and animals were randomized for experimental treatment.

### Isolation of primary RGC

Primary RGC were isolated following previously described protocols (Dvoriantchikova et al., 2014; Udeh et al., 2019; Musada et al., 2020). Retinas were dissected from postnatal day (P)10 to P12 mouse pups in 40 mm Petri dishes with Dulbecco PBS (DPBS). The isolated retinas were washed 3 times in DPBS and digested into a single cell suspension with an activated papain solution containing 16.5 U/ml papain (Worthington Biochemical Corp), 2 mg/ml L-cystine hydrochloride (Sigma-Aldrich) and 125 U/ml DNase (Sigma-Aldrich) in DPBS for 30 min at 37°C in 5% CO_2_. After gentle trituration, the activated papain was inhibited by adding 1.5 mg/ml ovo-mucoid (Sigma-Aldrich) solution and the retinal cell suspension was centrifuged at 1000 rpm for 10 min at room temperature. The pelleted retinal cells were mixed with immunopanning buffer (5 μg/ml insulin in DPBS) and the resulting retinal cell suspension was incubated with anti-macrophage antibody for 45 min on coated 150 mm Petri dishes to remove macrophages and endothelial cells. The suspended cells were then transferred into anti-Thy 1.2 antibody conditioned media (obtained from anti-mouse Thy 1.2 hybridoma cell culture) in coated Petri dishes and incubated for 45 min to isolate RGCs. The Petri dish was washed 8–10 times to remove other types of cells and then bound RGCs were removed by trypsinization and 50,000 RGCs were plated onto 15 mm cover slips placed in 24-well plates. RGCs were cultured in serum free neurobasal/B27 media (Thermo Fisher Scientific) at 37°C and 5% CO_2_. The purity of the RGC cultures was determined by immunodetection with anti-RBPMS antibody (PhosphoSolutions; 1:500) and were typically >95% pure.

To quantify primary RGC neurite growth and complexity, RGCs were treated for 48 h with Wnt5a (25–100 ng/ml) or BSA control (50 ng/ml), and then immunostaining was performed as described previously (Udeh et al., 2019; Musada et al., 2020). Cells were fixed in 4% paraformaldehyde, then permeabilized and blocked using 0.3% Triton X-100 and 10% goat serum. The coverslips were probed with primary rabbit anti‐βIII‐tubulin antibody (Abcam; 1:1000, AB18207, AB_444319) overnight at 4°C followed by Alexa Fluor 546-conjugated goat anti‐rabbit IgG secondary antibody (1:500, Invitrogen) for 1 h, and then mounted onto glass slides. Neurite number, neurite length and branch site number were measured in three biological replicates. In each replicate, neurites were measured from 100 to 200 RGC in at least three different random fields. Neurite length was measured for all the neurites longer than 5 μm using ImageJ software and then averaged. The number of primary, secondary and tertiary branch sites were counted manually and averaged and used as an indicator of neurite complexity. The treatment identity during imaging and measurements were masked to investigators.

### Optic nerve crush (ONC) injury and intravitreal injections

Male and female mice at age seven to eight weeks were randomly assigned to the treatment groups. ONC injury was performed as described previously (Park et al., 2008; Patel et al., 2017). The mice were anesthetized using an isoflurane mixture and one drop of 0.5% proparacaine hydrochloride was added to the eye. A small incision was made in the superior posterior area of the conjunctiva to expose the optic nerve. ONC was performed ∼1 mm behind the globe carefully avoiding blood vessels for 5 s with extra-fine self-closing forceps. The ONC eyes were intravitreally injected with recombinant Wnt5a (20 and 50 ng, in 2 μl; R&D Systems Inc) or the equivalent volume of sterile saline, using a 33-gauge Hamilton needle (Hamilton Company). Erythromycin ointment was topically applied to treat the injected eye and analgesia was provided with buprenorphine SR (0.5 mg/ml). Animals were excluded from the study if they had excessive bleeding or swelling at any time after the procedure.

### Axon quantification

Two days before the end of the experiment, the mice were intravitreally injected with 2 μg of Alexa Fluor 555-conjugated cholera toxin β subunit (CTB; Thermo Fisher Scientific) to label regenerating axons in the optic nerve. After euthanasia, the eyes and optic nerves were removed and fixed in 4% paraformaldehyde, processed in 5%, 10%, and 20% sucrose for 1 h each, and then embedded in OCT compound (Tissue Tek). Optic nerves were cryosectioned into 10 μm-thick longitudinal slices and the optic nerve sections were imaged using a fluorescent microscope (Zeiss). The number and the length of CTB-positive axons that extended past the crush site were counted as described (Park et al., 2008; Patel et al., 2017). At least four sections from each optic nerve were counted and averaged for each animal.

### Immunohistochemistry

Immunohistochemistry was performed on 10 μm-thick ocular cross-sections. The retinal sections mounted onto slides were probed with the RGC marker antibody anti-RBPMS (PhosphoSolutions, 1:250, #1832, RRID:AB_2492226) and with anti-Wnt5a (Santa Cruz, #SC-365370, RRID:AB_10846090) overnight at 4°C. The slides were washed three times in 1× PBS and then incubated with Alexa Fluor-conjugated secondary antibody (1:500; Invitrogen) for 1 h. After washing the slides again three times with PBS, sections were then covered with DAPI containing mounting medium (Vectashield) and imaged using a fluorescent microscope (Zeiss). Negative controls that lacked the primary antibody incubation were imaged at the same intensity settings to confirm lack of nonspecific staining by the secondary antibody. For RGC counts, RBPMS-positive/DAPI-positive cells were counted in the entire retina section in six sections per each animal and three animals per treatment group. Additionally, IHC was performed with antibodies specific to phospho (Thr286) CamKII (Cell Signaling Technology, catalog #12716, 1:200, RRID:AB_2713889), phospho (Thr183/Tyr185)-JNK (Abclonal, 1:200) or phospho (Ser129, Ser133) CREB (Thermo Fisher Scientific, 1:200, #44-297G, RRID:AB_2533625) following similar procedures.

### Western blotting

Retinas were isolated from experimental and control animals 1 d after intravitreal injection of 50 ng Wnt5a or saline into noninjured mice. Retinal lysates were prepared using RIPA buffer (Thermo Fisher Scientific) supplemented with protease and phosphatase inhibitors (Thermo Fisher Scientific). Fifteen micrograms of the protein samples were electrophoresed using 4–20% Mini-Protean TGX precast gels (Bio-Rad), and proteins were transferred to polyvinylidene fluoride (PVDF) membranes. The membranes were probed with antibodies specific to phospho (βII Ser660) protein kinase C (pPKC; Cell Signaling Technology; 1:1000, #9371S, RRID:AB_2168219), phospho (Thr286) CamKII (Cell Signaling Technology, 1:1000, #12716S, RRID:AB_2713889), phospho (Thr183/Tyr185)-JNK (Cell Signaling Technology, 1:1000, #4668S, RRID:AB_823588), total PKC (Cell Signaling Technology, 1:1000, #25453S, RRID:AB_2798904), total CamKII (Cell Signaling Technology, 1:1000, #4436S, RRID:AB_10545451), total JNK (Cell Signaling Technology, 1:1000, #9252S, RRID:AB_2250373), or GAPDH (Cell Signaling Technology, 1:1000, #5174S, RRID:AB_10622025) for 24 h at 4°C. The membranes were washed three times in PBS and probed with secondary antibody conjugated to horseradish peroxidase (1:500) for 1 h at room temperature. After additional washes, the membranes were incubated with enhanced chemiluminescence solution (Bio-Rad) and imaged. The protein bands were quantified using ImageJ software.

### RNA extraction and quantitative PCR

Total RNA was extracted from retinas isolated 3 d after ONC injury or from noninjured but anesthetized mice for endogenous *Wnt5a* measurements, and RNA was extracted from retinas after 1 d after intravitreal injection of 50 ng Wnt5a or saline from noninjured mice for *Stat3*, *Creb*, *Cntf*, *Atf3*, and *Pten* expression. RNA was extracted from retinal tissue using the RNeasy mini kit (QIAGEN) according to the manufacturer’s instructions and cDNA was prepared from 1 μg of RNA using iScript reverse transcription supermix (Bio-Rad). The QPCR reactions used PowerUP SYBR Green master Mix (Thermo Fisher Scientific) with three replicates per sample, and specific primers that span at least one intron/exon junction for *Wnt5a*, *Stat3*, *Creb*, *Cntf*, *Atf3*, and *Pten* are listed in Table 1. To determine the relative expression of the corresponding transcripts, the delta-delta Ct method was used with the calculations in https://toptipbio.com/delta-delta-ct-pcr using expression of the *ARP* gene as a reference housekeeping control gene.

**Table 1:** List of oligonucleotide primers used for qPCR

Gene name	Direction	Oligonucleotides 5'−3'
*Wnt5a*	Forward	CTCCTTCGCCCAGGTTGTTATAG
*Wnt5a*	Reverse	TGTCTTCGCACCTTCTCCAATG
*Stat3*	Forward	AATGGAAATTGCCCGGATCG
*Stat3*	Reverse	TCCTGAAGATGCTGCTCCAA
*Atf3*	Forward	TCTGCCATCCGATGTCCTCT
*Atf3*	Reverse	TTGTTTCGACACTTGGCAGC
*Cntf*	Forward	ATTCGTTCAGACCTGACTGC
*Cntf*	Reverse	CCTGATGGAAGTCACCTTCA
*Pten*	Forward	CCCAGTCTCTGCAACCATCCAG
*Pten*	Reverse	AGTCTTTCTGCAGGAAATCCCAT

### Statistical analysis

Statistical analyses were performed using GraphPad Prism (GraphPad Software, Inc). Each animal was considered as an individual data point. Normality tests were performed on all datasets. Multiple comparison analyses (Figs. 3-5) were conducted using ANOVA with Tukey’s *post hoc* tests. Comparisons with only two groups (Figs. 2, 6–7, 11) were performed using Student’s unpaired *t* tests; *p* values <0.05 were considered statistically significant. Sample sizes are listed in the figure legends.

## Results

### Wnt5a is expressed in adult mouse retina and is upregulated after injury

**Figure 1.**
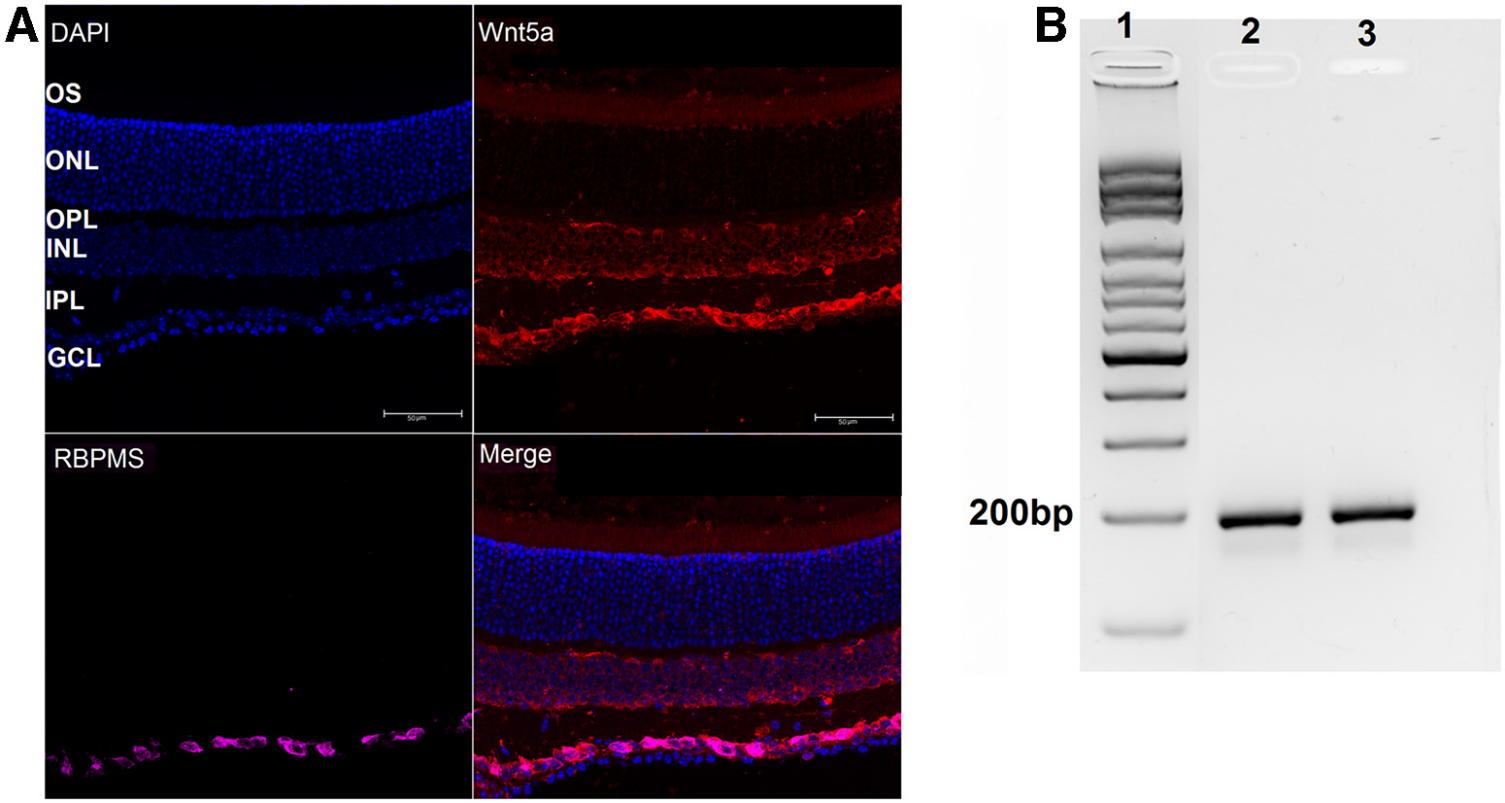
Wnt5a is expressed in RGCs. ***A***, Expression of Wnt5a in adult mouse retina. DAPI-stained nuclei (blue) indicate the retinal layers. Wnt5a expression (red) was observed in GCL, INL, and OS layers and Wnt5a was colocalized with the RGC marker RBPMS (pink, colocalization is shown as magenta) in the GCL (GCL: ganglion cell layer, IPL: inner plexiform layer, INL: inner nuclear layer, OPL: outer plexiform layer, ONL: outer nuclear layer and OS: outer segment layer). Scale bar: 50 μm. ***B***, Amplified Wnt5a PCR product from cDNA prepared from total RNA of RGC primary cultures. Lane 1: ladder; lanes 2 and 3: 196 bp amplified Wnt5a PCR product from two different RGC cultures.

Previous reports show dynamic Wnt5a expression in the developing and adult retina. Wnt5a decreases after development is complete (Liu et al., 2003) and is upregulated in postnatal retinas during photoreceptor degeneration (Yi et al., 2007). To determine the baseline expression pattern of Wnt5a in adult mouse retina, we probed retinal sections from six-week-old mice with an anti-Wnt5a antibody. As shown in Figure 1, Wnt5a was detected in the ganglion cell layer (GCL), inner nuclear layer (INL) and photoreceptor outer segment layers (Fig. 1*A*). No fluorescent signal was observed in control retinal sections stained without primary antibody (data not shown). Co-detection of Wnt5a with the RGC marker RBPMS indicated that the majority of Wnt5a in the GCL is expressed in RGCs (Fig. 1*A*). The expression of Wnt5a in RGCs was also confirmed in primary RGCs using PCR (Fig. 1*B*). Furthermore, localization of Wnt5a in the INL is consistent with expression of Wnt5a transcripts in primary Muller glia cultures (Musada et al., 2020).

**Figure 2.**
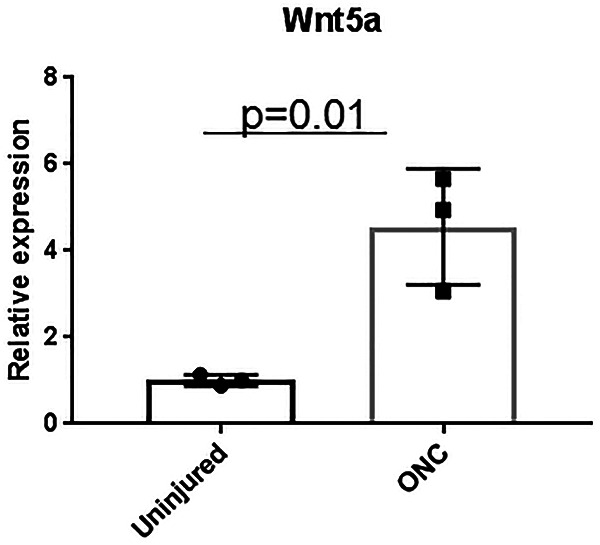
Upregulation of endogenous Wnt5a after ONC injury. QPCR quantification of Wnt5a gene expression 3 d after ONC injury showed significant upregulation in retinas from ONC injured mice compared with retinas from uninjured but anesthetized mice (*n* = 3). Mean ± SD is shown. See Extended Data 1 for detailed statistical analyses.

To determine whether Wnt5a levels in the retina change after ONC injury, we measured Wnt5a transcript levels using quantitative PCR in retinas obtained 3 d after ONC, which is the time point at which RGC death is evident (Yasuda et al., 2016). This analysis showed that Wnt5a levels were upregulated >3-fold (*p* = 0.01) in ONC injured retinas compared with uninjured retinas (Fig. 2). Therefore, endogenous Wnt5a is upregulated after RGC injury, similar to the findings in retinas with degenerating photoreceptors (Yi et al., 2007).

10.1523/ENEURO.0182-22.2022.ed1 Extended Data 1 Statistics information Identification of a Novel Axon Regeneration Role for Non-Canonical Wnt Signaling in the Adult Retina After Injury. Download Extended Data 1, DOCX file.

### Wnt5a induces RGC neurite growth and increases neurite complexity

**Figure 3.**
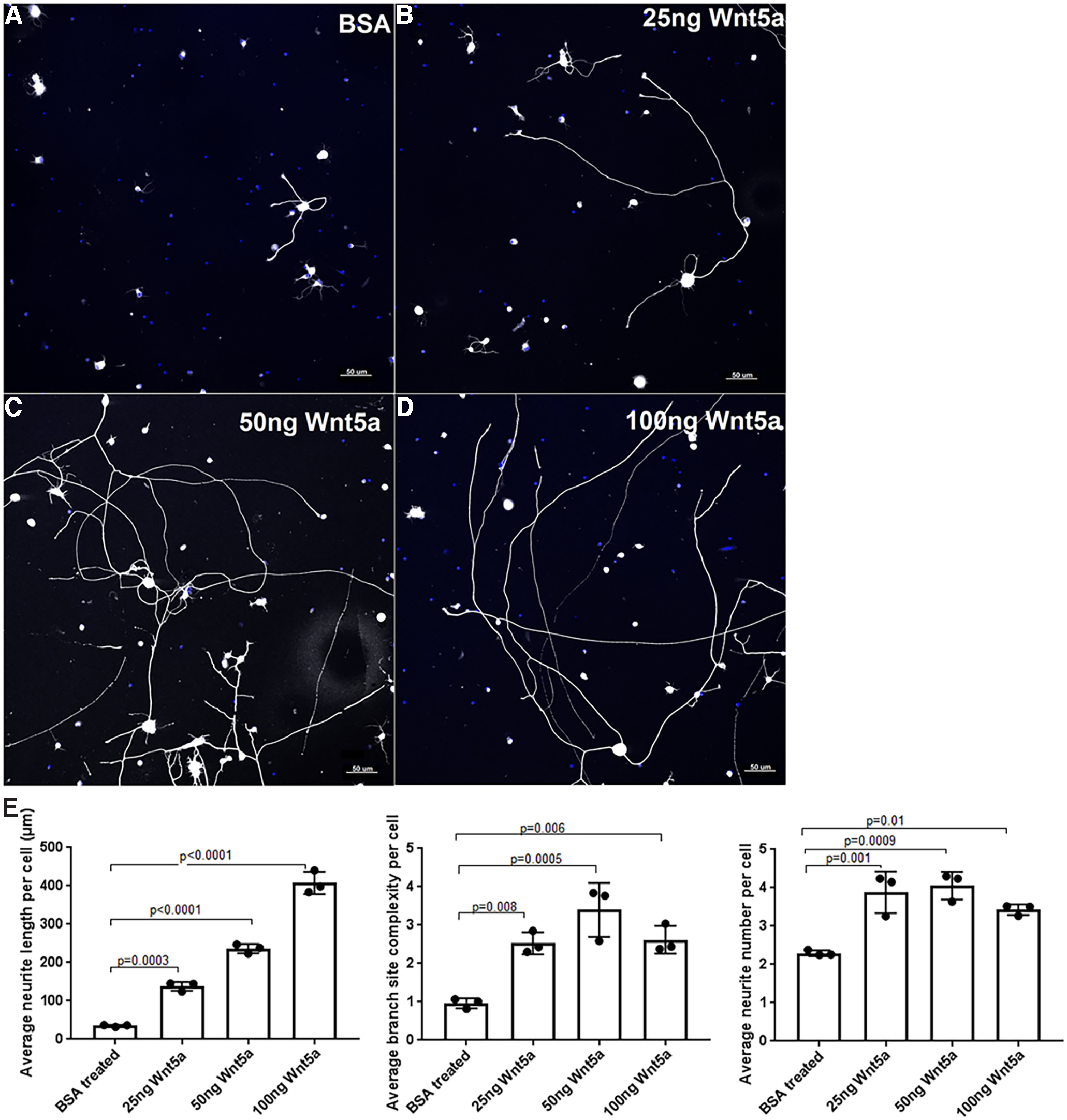
Wnt5a treatment induced neurite growth, neurite complexity and neurite number in RGC primary cultures. ***A–D***, Representative images of RGC cultures treated with BSA (control), 25, 50, and 100 ng recombinant Wnt5a. Neurites are shown in white, DAPI-stained nuclei are blue. Scale bar: 50 μm. ***E***, Bar diagrams show significantly increased average RGC neurite length, ANC (branch site number) and ANN in different concentrations of Wnt5a-treated cultures compared with BSA-treated cultures. Mean ± SD is shown. See Extended Data 1 for detailed statistical analyses.

To identify potential activities of Wnt5a in RGCs, we first investigated the effect of exogenous Wnt5a on RGC neurite growth. Primary RGC cultures were treated with BSA control or 25 ng/ml, 50 ng/ml or 100 ng/ml recombinant Wnt5a. As shown in Figure 3, Wnt5a significantly increased neurite length, neurite complexity (branch site number) and neurite number. The average neurite length (ANL) per cell in the BSA-treated group was 35.16 ± 2.63 μm, whereas the ANL of 25, 50, and 100 ng of Wnt5a groups were 138 ± 11.6, 236.5 ± 11.9, and 407.6 ± 29.5 μm, respectively. A dose-effect was also demonstrated in which significantly longer neurites were observed with increasing Wnt5a concentration: 25 versus 50 ng (*p* = 0.0005), 50 versus 100 ng (*p* < 0.0001), and 25 versus 100 ng (*p* < 0.0001). Additionally, the highest average neurite complexity (ANC) and average neurite number (ANN) were observed in the 50-ng treatment compared with BSA: the ANC in Wnt5a 25, 50, and 100 ng groups were 2.527 ± 0.29, 3.4 ± 0.7, and 2.62 ± 0.3, respectively, compared with the BSA control which was 0.9 ± 0.1. The ANN was increased in all Wnt5a treatments although increasing concentration did not enhance it further: the ANN for the 25, 50, and 100 ng Wnt5a-treated cultures were 3.8 ± 0.5, 4.06 ± 0.36, and 3.4 ± 0.1 compared with the BSA control that had 2.29 ± 0.07 (Fig. 3). Therefore, these data indicate that Wnt5a stimulates dose-dependent neurite growth and neurite complexity and increases neurite number in RGC cultures.

### Wnt5a protects RGCs after optic nerve injury

**Figure 4.**
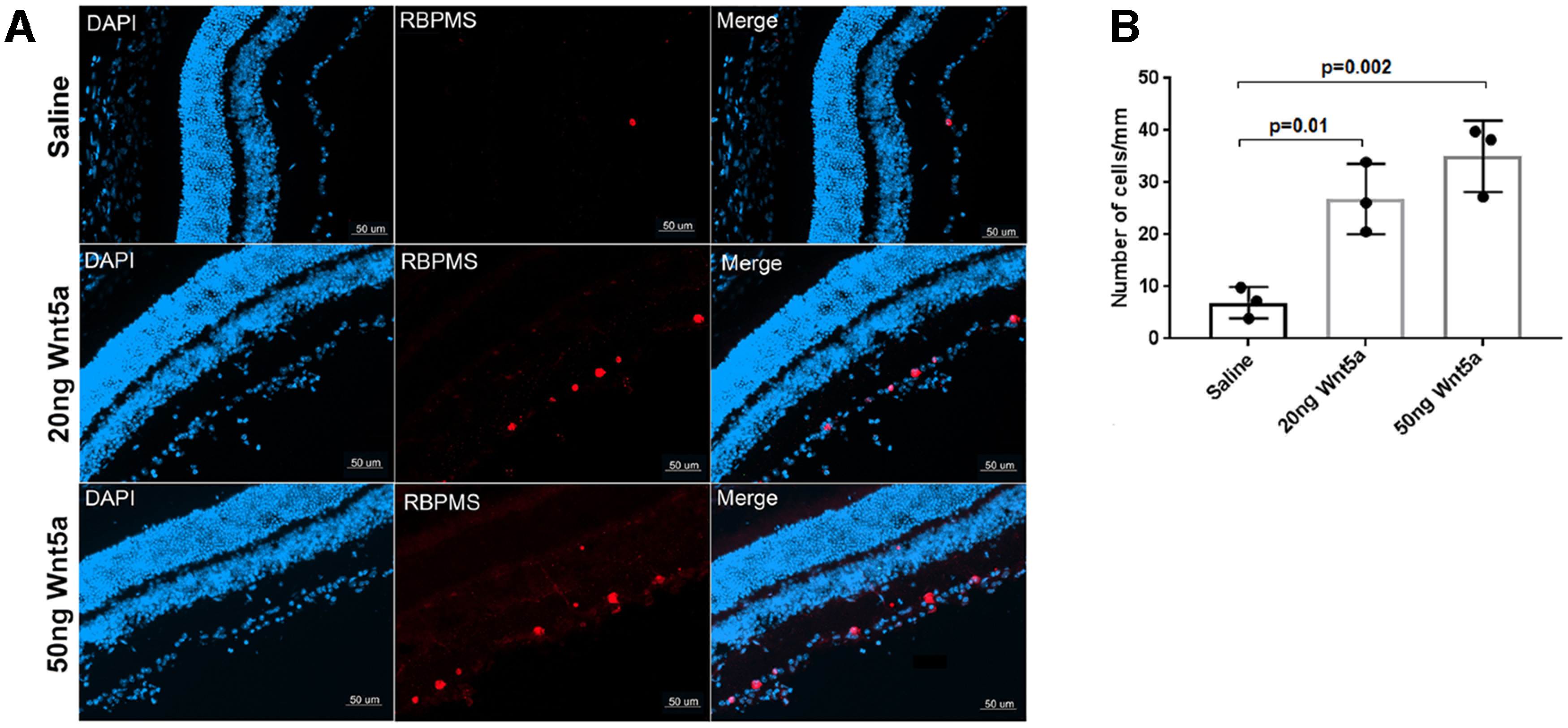
Wnt5a protects RGCs after ONC injury. ***A***, Immunohistochemistry on retinal cryosections two weeks after ONC injury showed increased RBPMS-positive RGCs (red) in 20 and 50 ng Wnt5a-injected retinas compared with saline-injected retinas. DAPI-stained nuclei (blue) indicate the retinal layers. Scale bar: 50 μm. ***B***, Quantification of RBPMS immunopositive cells that colocalized with DAPI shows significantly increased RGC density in 20 and 50 ng Wnt5a-injected retinas compared with saline-injected retinas. Mean ± SD is shown. See Extended Data 1 for detailed statistical analyses.

Next, we investigated the effect of recombinant Wnt5a protein in vivo. First, RGC survival after ONC injury was measured by detecting the number of remaining RGCs two weeks after ONC in Wnt5a-injected or saline-injected retinas. This analysis demonstrated that Wnt5a has a significant effect on RGC survival. Whereas only 6.9 ± 3 cells/mm were observed in the saline-injected group, 26.8 ± 6.7 cells/mm were observed in the 20 ng Wnt5a-injected group (*p* = 0.01 vs saline) and 35.0 ± 6.8 cells/mm (*p* = 0.002 vs saline) in the 50 ng Wnt5a group (Fig. 4). Therefore, Wnt5a induces survival of RGCs after optic nerve injury.

### Wnt5a promotes axonal regrowth after ONC

**Figure 5.**
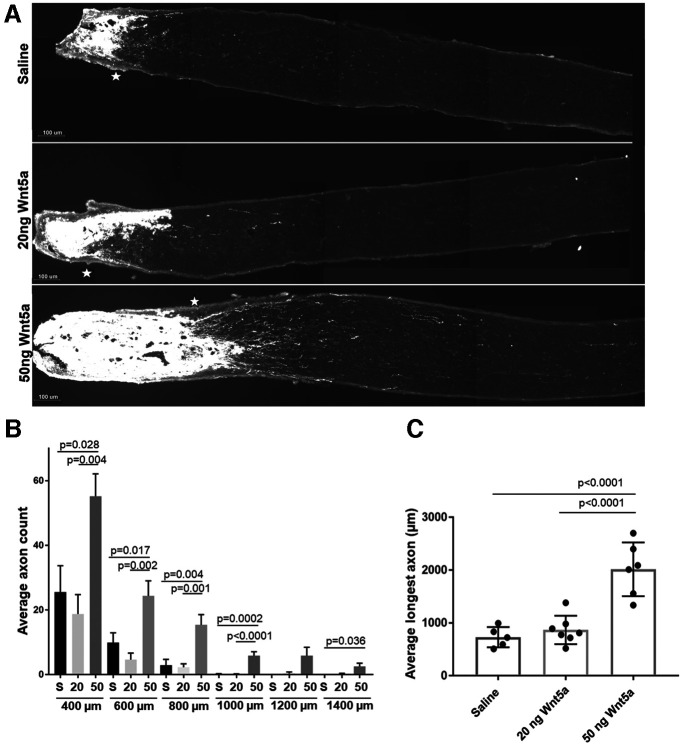
Axonal regeneration two weeks after ONC injury following a single intravitreal injection of Wnt5a. ***A***, Representative images of CTB-labeled axons (white) demonstrating increased axons past the crush region (*) in 50 ng Wnt5a-injected mice compared with saline-injected or 20 ng Wnt5a-injected mice. Scale bar: 100 μm. ***B***, Average axon count for each distance past the crush site (400–1400 μm) demonstrates significantly higher regeneration in 50 ng Wnt5a-injected animals compared with saline and 20 ng Wnt5a-injected animals (mean ± SEM). ***C***, Comparison of the longest axon detected per nerve for each mouse shows significantly longer regrowth in the 50 ng Wnt5a-injected animals compared with saline and 20 ng Wnt5a-injected animals (mean ± SD). S = saline, 20 = 20 ng Wnt5a, 50 = 50 ng Wnt5a; *n* = 5 or 6 animals per each group. See Extended Data 1 for detailed statistical analyses.

Next, we examined whether Wnt5a promoted axonal regeneration after ONC injury. The length and number of axons in the optic nerve were quantified using CTB to label axon regrowth past the injury site. Retinas injected with 50 ng Wnt5a showed significantly longer axons (Fig. 5*A*) and higher average axon counts (Fig. 5*B*) compared with saline-injected eyes when measured two weeks after ONC injury. Quantifying axon growth at specific distances from the crush site assessed at 200 μm intervals shows significantly more axons in the 50 ng Wnt5a-treated group at all sites (400–1400 μm) compared with saline (Fig. 5*B*). Whereas the majority of axons in the saline-injected mice grew 400 μm or less and very few grew past 800 μm, the axons in the Wnt5a group grew to 1400 μm (Fig. 5*B*). Furthermore, we quantified the average longest axon in each nerve to indicate the maximum regrowth of axons after Wnt5a injection compared with control. Although it is possible that a longer axon may have been missed (for example, if it was close to the edge of the nerve), this analysis found that the average length in all the 50 ng Wnt5a-injected mice was significantly higher than control. Injection of 20 ng Wnt5a did not promote axonal growth after ONC injury, in contrast to its effect on RGC survival and neurite growth in culture. Therefore, these results demonstrate that the 50 ng dose of Wnt5a induced significant axonal regeneration after injury.

### Wnt5a increased phosphorylation of CamKII/CREB and JNK and decreased phosphorylation of PKC

**Figure 6.**
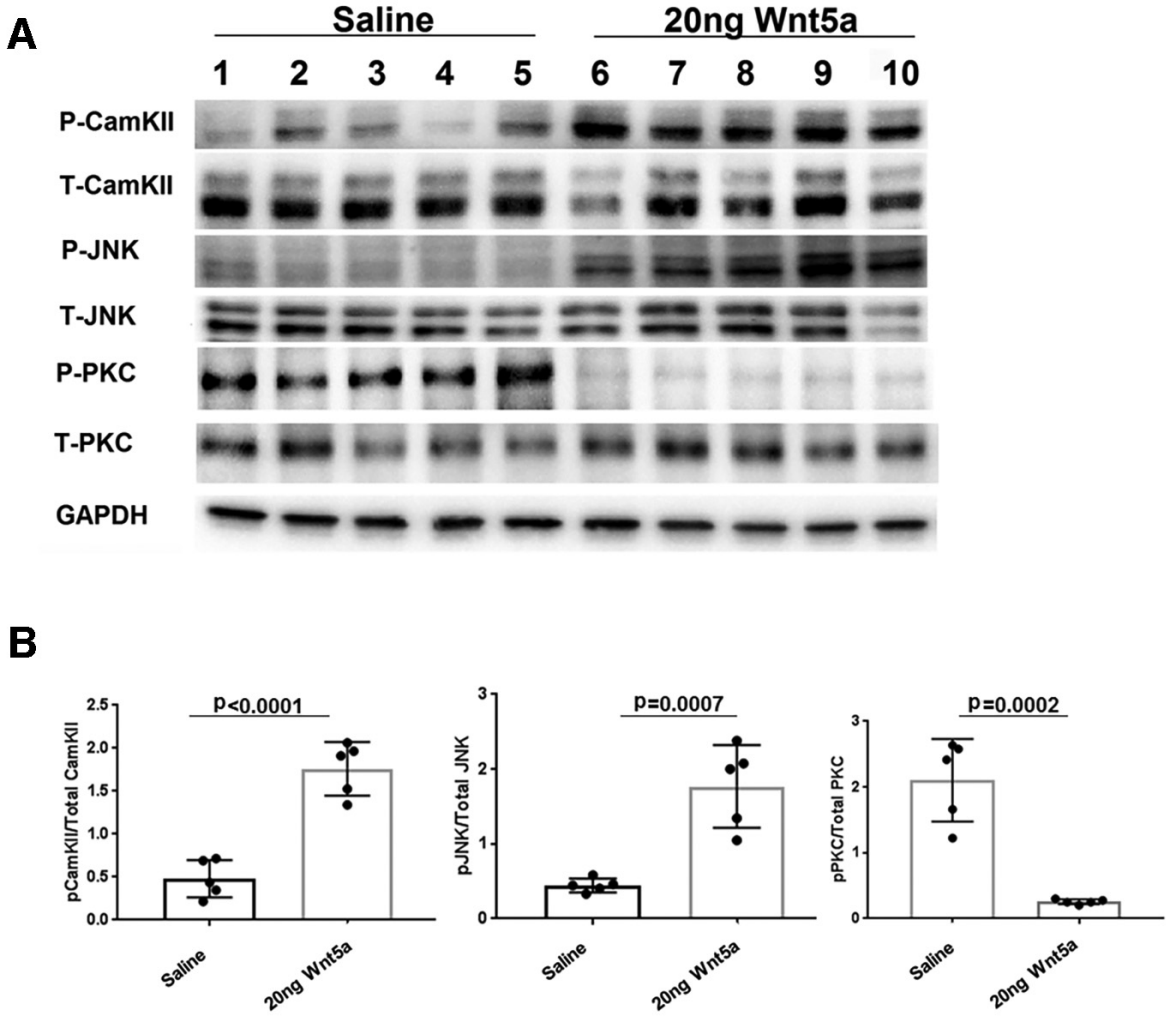
CamKII and JNK signaling is upregulated and PKC signaling is downregulated by Wnt5a. ***A***, Representative Western blot images of phospho and total CamKII, phospho and total JNK, phospho and total PKC, and GAPDH on retinas collected 24 h after Wnt5a or saline injection. ***B***, Normalized phospho protein band intensity with total protein band intensity of CamKII, JNK, and PKC shows significant upregulation of phospho CamKII and JNK in 20 ng Wnt5a-injected retinas compared with saline-injected retinas. In contrast, significant downregulation of phospho PKC was observed in 20 ng Wnt5a-injected retinas compared with the saline-injected retinas (*n* = 5 in each group). Mean ± SD is shown. See Extended Data 1 for detailed statistical analyses.

**Figure 7.**
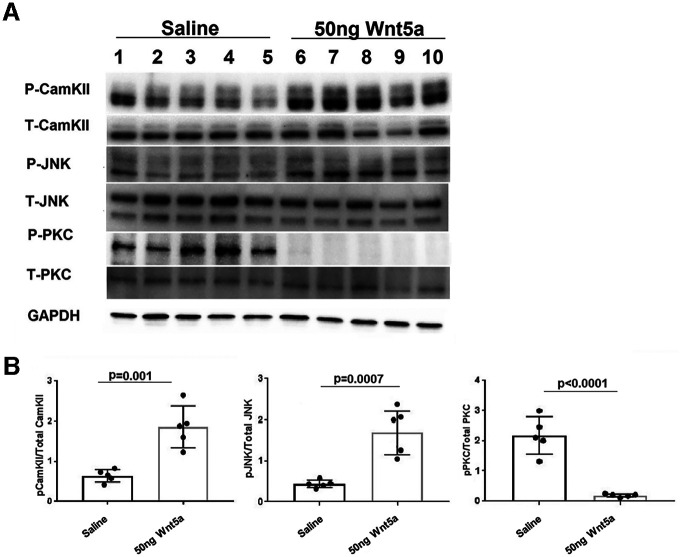
CamKII and JNK signaling is upregulated by Wnt5a and PKC signaling is downregulated by Wnt5a. ***A***, Representative Western blot images of phospho and total CamKII, phospho and total JNK, phospho and total PKC, and GAPDH. ***B***, Normalized phospho protein band intensity with total protein band intensity of CamKII, JNK, and PKC shows significant upregulation of phospho CamKII and JNK in 50 ng Wnt5a-injected retinas compared with saline-injected retinas. In contrast, significant downregulation of phospho PKC was observed in 50 ng Wnt5a-injected retinas compared with the saline-injected retinas (*n* = 5 in each group). Mean ± SD is shown. See Extended Data 1 for detailed statistical analyses.

To investigate signaling mechanisms induced by Wnt5a in the retina, we quantified levels of specific activated kinases downstream of Wnt5a using antibodies against their phosphorylated forms. Wnt5a primarily signals through activation of CamKII, PKC, and JNK, suggesting that these kinases are good candidates for mediating the effects of Wnt5a in the retina. Because Wnt5a induces its downstream signaling mediators within 10–20 min of addition to cultured cells (Li et al., 2009), we collected retinas 24 h after intravitreous injection to provide enough time for Wnt5a to diffuse through the vitreous and induce signaling in retinal cells. In order to measure stimulation of these pathways from only Wnt5a, the optic nerves were not injured in these experiments. As shown in Figures 6, 7, Western blotting demonstrated greater than a 2-fold increase of the phosphorylated forms of CamKII and JNK in Wnt5a-injected retinas compared with saline injections (*p* < 0.05). In contrast, Wnt5a injections reduced the phosphorylation of PKC >4-fold compared with saline-injected eyes (*p* < 0.05).

**Figure 8.**
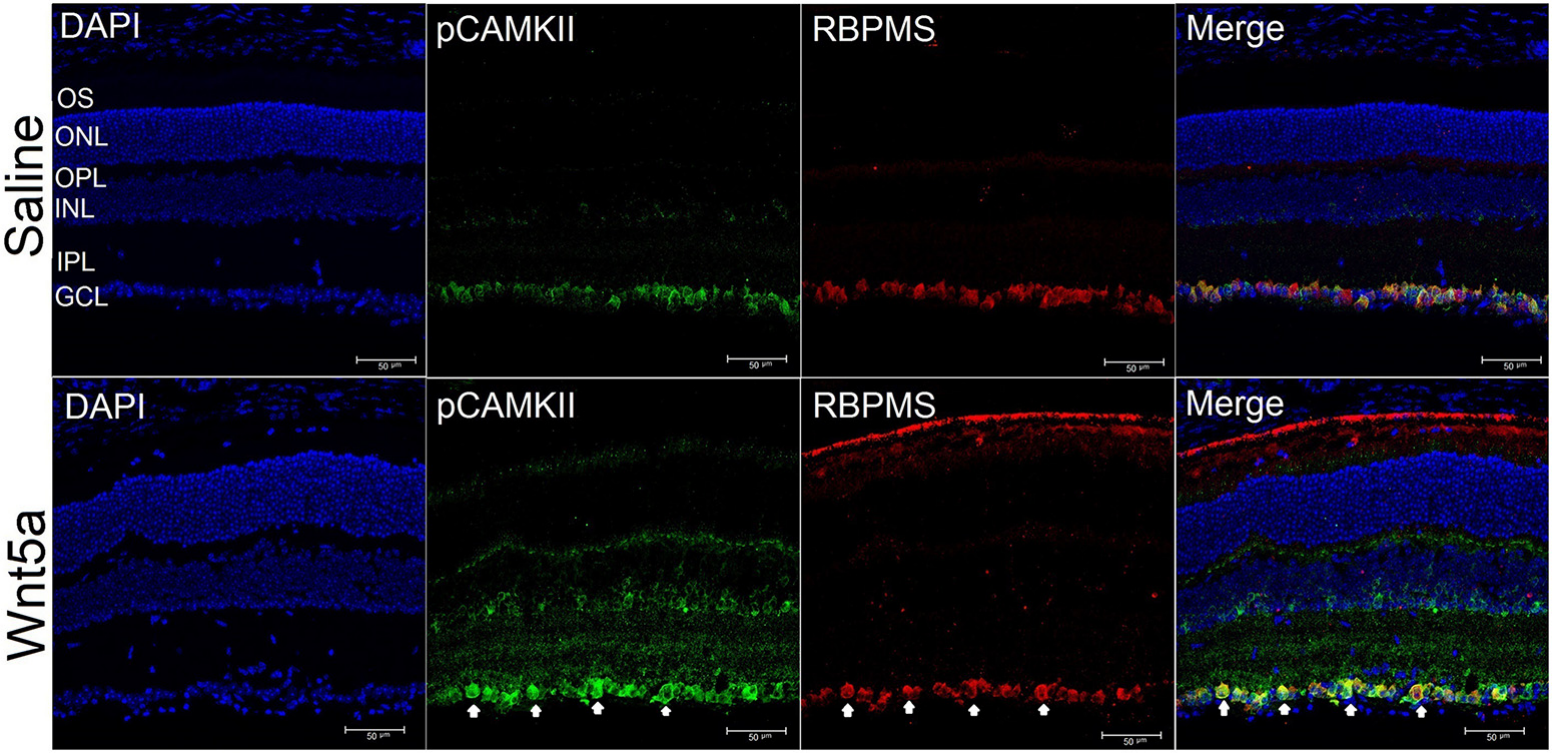
Increased phosphorylation of CamKII in RGCs 24 h after a Wnt5a injection. Upper panel shows representative images of cross-sections of saline-injected retinas. Lower panel shows representative images of cross-sections of 50 ng Wnt5a-injected retinas. IHC was performed to co-immunostain phospho CamKII (green) and RBPMS (red). Phosphorylation of CamKII was induced in GCL, INL, and OPL of Wnt5a-injected retinas compared with saline-injected retinas (GCL: ganglion cell layer, IPL: inner plexiform layer, INL: inner nuclear layer, OPL: outer plexiform layer, ONL: outer nuclear layer and OS: outer segment layer). Colocalization of CamKII phosphorylation with RBPMS-positive cells is indicated by yellow overlap signal (white arrows). Scale bar: 50 μm.

**Figure 9.**
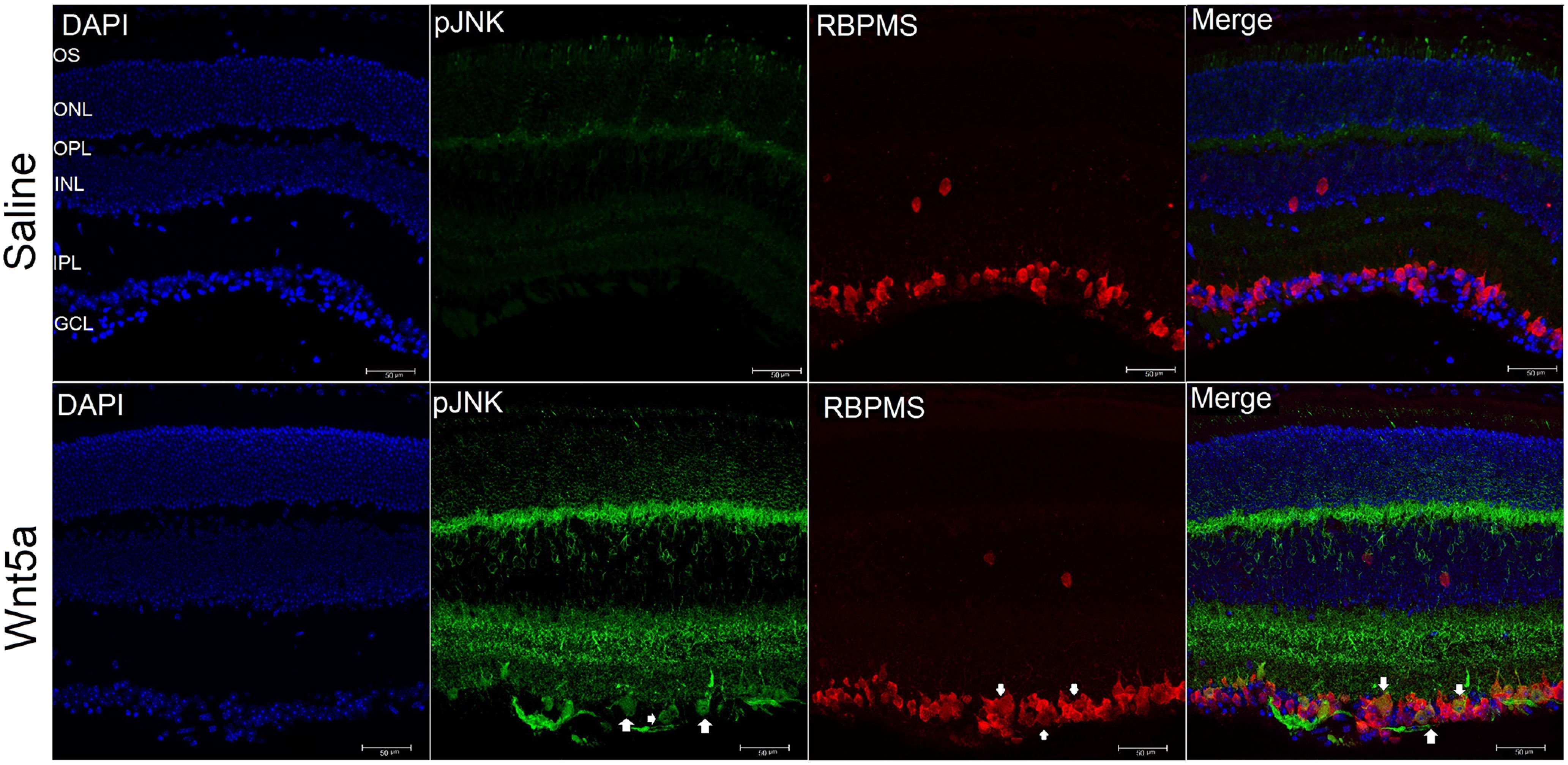
Increased phosphorylation of JNK in Wnt5a-injected retinas. Upper panel shows representative images of cross-sections of saline-injected retinas. Lower panel shows representative images of cross-sections of 50 ng Wnt5a-injected retinas. IHC was performed to co-immunostain phospho JNK (green) and RBPMS (red). Phosphorylation of JNK was induced in certain cell somas of RGC, IPL, and OPL in Wnt5a-injected retinas compared with saline-injected retinas (GCL: ganglion cell layer, IPL: inner plexiform layer, INL: inner nuclear layer, OPL: outer plexiform layer, ONL: outer nuclear layer and OS: outer segment layer). White arrows indicate induced phosphorylation of JNK that colocalized with RBPMS-positive RGCs. Scale bar: 50 μm.

**Figure 10.**
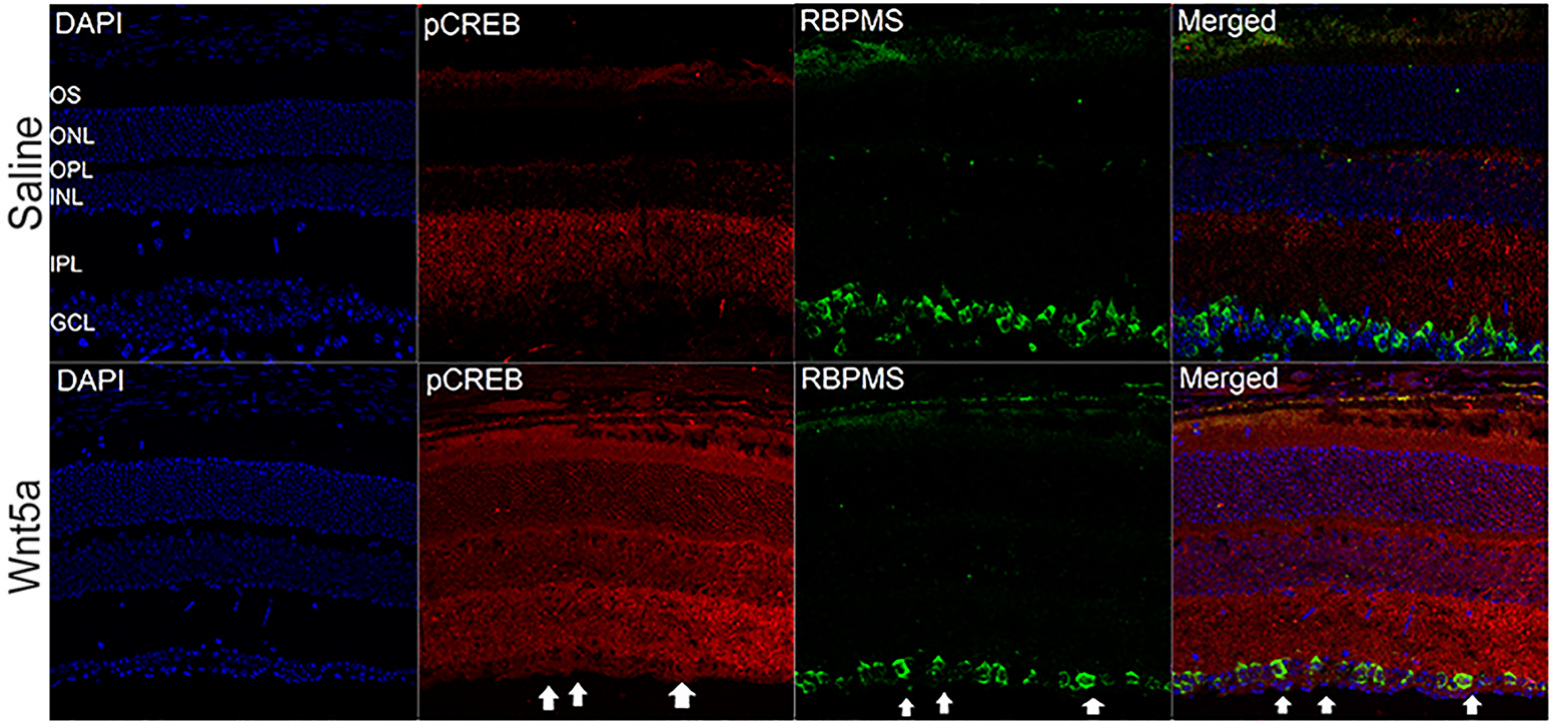
Increased phosphorylation CREB in Wnt5a-injected retinas. Upper panel shows representative images of cross-sections of saline-injected retinas. Lower panel shows representative images of cross-sections of 50 ng Wnt5a-injected retinas. IHC was performed to co-immunodetect phospho CREB (red) and RBPMS (green). Phosphorylation of CREB was induced in the GCL and other layers of the retina. White arrows indicate induced phosphorylation of CREB in RBPMS-positive RGC cell somas (GCL: ganglion cell layer, IPL: inner plexiform layer, INL: inner nuclear layer, OPL: outer plexiform layer, ONL: outer nuclear layer and OS: outer segment layer). Scale bar: 50 μm.

Next, because the Western blottings were performed on whole retinas and RGCs are a small portion of total cells in the retina, we used immunohistochemistry on retinal sections to identify whether RGCs expressed the activated signaling kinases, using antibodies specific to phospho CamKII, phospho JNK and phospho CREB. These experiments showed greater detection of the phosphorylated forms of CamKII (Fig. 8), JNK (Fig. 9), and CREB (Fig. 10) that codetected with the RGC-specific protein RBPMS in Wnt5a-injected eyes compared with saline-injected eyes, indicating activation of these kinases in RGCs. In addition to RGCs, the phosphorylation of CamKII and JNK was also observed in the INL in a pattern consistent with Muller glia. Furthermore, phosphorylation of CamKII but not JNK was observed in non-RGCs as well as RGCs in the GCL.

### Wnt5a induces multiple prosurvival and proregenerative pathways

**Figure 11.**
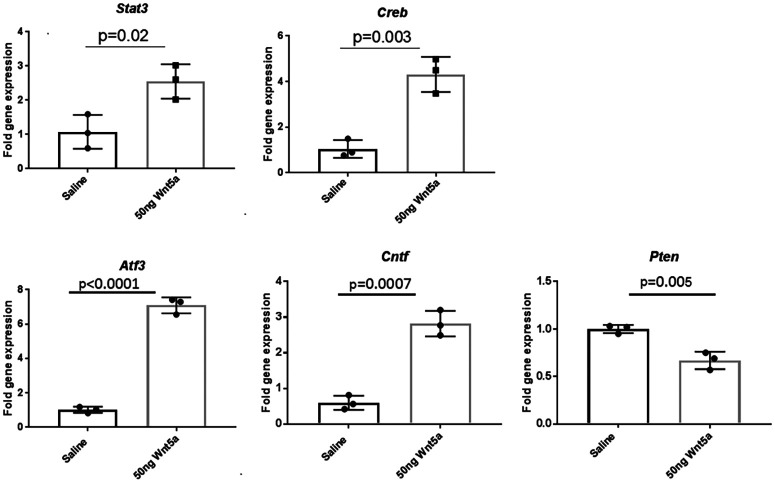
QPCR quantification of *Stat3*, *Creb*, *Atf3*, *Cntf*, and *Pten* gene expression in retinas 1 d after intravitreal injections of 50 ng Wnt5a or saline (*n* = 3). Bar diagrams shows significant upregulation of *Creb*, *Stat3*, *Atf3*, and *Cntf* genes in Wnt5a-injected retina compared with saline-injected retinas. Significant downregulation of *Pten* gene expression was observed in Wnt5a-injected retinas compared with saline-injected retinas. Mean ± SD is shown. See Extended Data 1 for detailed statistical analyses.

We next measured expression levels of transcription factors *Stat3* and *Creb*, which are stimulated by JNK and CamKII, respectively, in eyes injected with Wnt5a and saline and collected 24 h after injection. As with the phospho-protein analysis above, the nerves were not injured in these experiments to measure stimulation of the pathways from only Wnt5a. In Wnt5a-injected eyes, we observed >2-fold upregulation of *Stat3* and >3-fold upregulation of *Creb* by QPCR compared with saline-injected eyes, supporting activation of these signaling pathways. Additionally, it was previously reported that CREB induces *Atf3* and *Cntf* expression (Moore and Goldberg, 2011; Modi et al., 2013). Similarly, in Wnt5a-injected eyes, we demonstrated >3-fold upregulation of *Atf3* and *Cntf* compared with saline-injected eyes (Fig. 11). Finally, silencing of Wnt5a mRNA was reported to increase expression levels of *Pten* in Schwann cells (Yu et al., 2018). Consistent with that finding, we observed significant downregulation of *Pten* expression levels of Wnt5a-injected eyes compared with control.

## Discussion

Adult RGCs have very poor regenerative abilities after an axonal injury similar to other CNS neurons. Molecules and signaling mechanisms that induce RGC survival, RGC axon growth and optic nerve regeneration have potential to treat optic neuropathies and are a major topic of investigation in the field (Garcia et al., 2018). Although it was reported that noncanonical Wnt signaling pathways play multiple roles in CNS and retinal development (Liu et al., 2003; Arenas, 2014; Subashini et al., 2017; Xiao et al., 2017; He et al., 2018; Morenilla-Palao et al., 2020), the functions of noncanonical Wnt ligands in adult retina have not been characterized. In the present study, we investigated the role of the noncanonical Wnt ligand Wnt5a in the retina after optic nerve injury and investigated its potential mechanisms of action. Our findings demonstrated that Wnt5a regulated JNK, CamKII, and PKC signaling pathways in RGCs and other retinal cell types. Furthermore, Wnt5a led to significant RGC protection and optic nerve regrowth after optic nerve injury. This study demonstrated for the first time that Wnt5a regulates adult RGC survival, RGC axon growth and optic nerve regeneration and identified potential contributing signaling pathways.

We observed that Wnt5a significantly increased neurite length, neurite branch site number (complexity) and neurite number in primary RGC cultures. An important caveat is that the RGCs were obtained from young postnatal animals because of the technical limitations of culturing RGCs from adult retinas. The effects we observed of Wnt5a on RGC neurites are corroborated by previous reports on different types of CNS neurons. For example, Wnt5a enhanced neurite growth and complexity through activating noncanonical Wnt pathways in cultures of olfactory bulb interneurons (Pino et al., 2011), induced neurites on rod photoreceptors but not cone photoreceptors in retina cultures (Sarin et al., 2018) and enhanced neurite growth and complexity of cortical neuron and dopaminergic neurons in the brain (Li et al., 2009; Blakely et al., 2011; Hutchins et al., 2011). This function is also observed during visual system development in that Wnt5a guides RGC axon growth and promotes midline crossing of contralateral RGC axons (Morenilla-Palao et al., 2020). Additionally, Wnt5a induced axon growth and neurite branch extension in sympathetic neuron cultures that was transcription and translation independent and required Wnt5a-induced regulation of cytoskeletal effectors (Bodmer et al., 2009).

In contrast, Miyashita et al. (2009) reported that Wnt5a inhibited neurite outgrowth in cerebellar granule neurons through RYK receptor mediated signaling mechanisms (Miyashita et al., 2009). The authors demonstrated that inhibiting Wnt5a/RYK signaling using a neutralizing antibody induced corticospinal tract axon growth from the spinal cord injury site. A repulsive effect of Wnt5a was observed in axons of cortical and dopaminergic neurons that was mediated by RYK receptor activation (Li et al., 2009; Blakely et al., 2011). Also, although Wnt5a enhanced axonal growth of embryonic contralateral RGC axons it inhibited neurite growth and caused growth cone collapse of ipsilateral RGC axons, indicating differential responses to Wnt5a in different RGC subtypes (Morenilla-Palao et al., 2020). Gradients of Wnt5a have been shown to be critical for guiding axons during the developing CNS. For example, decreasing mediolateral gradients of Wnt5a during development steers axons through the intermediate zone within the contralateral hemisphere of the brain (Keeble et al., 2006), and Wnt5a gradients repel cortical axons into developing callosal and corticospinal pathways (Li et al., 2009). The molecular basis for neurite outgrowth and axon guidance or repulsive effects of Wnt5a in the developing visual system were recently reported to involve β-catenin, EphB1 receptors and the Zic2 transcription factor (Morenilla-Palao et al., 2020), but it remains to be identified whether axonal regrowth in adult RGCs also involves these molecular pathways.

We also demonstrated that Wnt5a induced a significant protective effect on RGCs after ONC injury. These findings are supported by previous studies demonstrating that Wnt5a prevented apoptosis and protected cortical neurons challenged with β-amyloid protein (Zhou et al., 2017) and Wnt5a downregulated cyclin D1, regulated cell cycle reentry and reduced apoptosis of cortical neurons (Zhou et al., 2017). Wnt5a also induced survival of dopaminergic neurons and human umbilical venous endothelial cells (HUVEC; Masckauchán et al., 2006; Blakely et al., 2011). We also observed that endogenous Wnt5a was upregulated after optic nerve injury, similar to reported findings of upregulated Wnt5a after photoreceptor degeneration (Yi et al., 2007). Future gene knock-down studies will be required to determine whether endogenous Wnt5a has a protective function after injury similar to the protective effect shown for exogenous Wnt5a.

Similar to cortical, sympathetic and hippocampal neurons (Bodmer et al., 2009; Farías et al., 2009; Hutchins et al., 2011), we observed activation of CamKII and JNK in RGCs after Wnt5a injections in the mouse retina. Interestingly, both 20 and 50 ng regulate JNK, CamKII, and PKC and promote RGC survival, but only the 50 ng dose promotes axonal growth. Therefore, our study identified a Wnt5a dose that distinguishes survival from axon regeneration mechanisms, and future studies will determine whether activation of these kinases by Wnt5a are sufficient for RGC survival but require stimulation of additional pathways for regeneration. Also, the optic nerves were not injured in these experiments to allow us to detect stimulation of these pathways from Wnt5a alone. Therefore, our findings identified CamKII, JNK, and PKC as signaling pathways regulated by Wnt5a but confirmation that these kinases are associated with the regenerative and/or neuroprotective effects of Wnt5a in the retina will require specific pathway inhibitors and cell-specific gene knock-outs.

Our results suggest that CamKII and JNK are candidate mediators of the neuroprotective effects of Wnt5a on RGCs. Previous studies demonstrated a role for different isoforms of JNK in neuroprotection and axon regeneration, which lends support for further investigators of JNK signaling as a potential contributor to the protective effects of Wnt5a (Waetzig et al., 2006; Atkinson et al., 2011; Tönges et al., 2011). However, it was also reported that certain JNK isoforms induce proapoptotic pathways in CNS neurons during excitotoxic stress, nerve transection and various pathologies such as Parkinson’s disease (Waetzig et al., 2006; Tönges et al., 2011). In addition to activating downstream transcription factors, JNK also induces axon regeneration by stabilizing cytoskeletal proteins (Barnat et al., 2010). Therefore, it will be interesting to determine whether stabilized cytoskeletal molecules from JNK activation play a role in RGC axon regeneration. Additionally, we observed increased expression of the transcription factors CREB and STAT3, which mediate CamKII and JNK signaling, respectively, after intravitreal injections of Wnt5a. Although currently shown by gene expression changes in uninjured retinas, these data provide potential mechanisms of action for Wnt5a because STAT3 is known to mediate axonal regeneration and survival from Wnt3a-induced signaling (Patel et al., 2017) and other experimental treatments (Leibinger et al., 2013; Luo et al., 2016). Additionally, we showed increased ATF3 after Wnt5a injection; ATF3 is a downstream target of JNK and CREB signaling and its overexpression was previously correlated with peripheral axon regeneration in the mouse sciatic nerve after crush injury (Seijffers et al., 2006; Zhang et al., 2011; Fagoe et al., 2015) and regeneration of RGC axons in the mouse optic nerve (Kole et al., 2020). Furthermore, Guo et al. (2021) reported that CamKII/Creb signaling protects RGCs and induces optic nerve regeneration in adult mice after excitotoxic NMDA treatment or axonal injuries, and transduction of a constitutively active form of CamKII (T286D) into adult RGCs provides long-term protection to RGC and their axons in glaucoma and ONC injury mouse models (Guo et al., 2021). Therefore, further investigation is warranted to determine whether Wnt5a-induced CamKII/CREB and JNK/Stat3 signaling, possibly through Atf3, contributes to RGC protection and optic nerve regeneration.

Along with increased phosphorylation of CamKII and JNK, Wnt5a injections also led to decreased phosphorylation of PKC in retinas. Although this finding may reflect a difference between injury and noninjury conditions or are specific to the time point after Wnt5a delivery, PKC was shown to contribute to the inhibitory effects of myelin and chondroitin sulfate proteoglycans on neurite outgrowth of rat CGNs (Sivasankaran et al., 2004), suggesting PKC inhibition may be relevant to Wnt5a-induced axonal growth. In contrast, PKC isoforms were upregulated in regenerating peripheral nerves, CNS and spinal cord neurons (Campenot et al., 1994; Wu et al., 2003; Tsai et al., 2007; Bodmer et al., 2009; Yang et al., 2010). PKC activators such as phorbol esters and bryostatin-1 induced neuronal survival and neurite growth in a PKC-βI-dependent manner in spiral ganglion neurons (Lallemend et al., 2005) and PKC inhibitors reduced regenerative axonal growth in PNS and CNS neurons (Campenot et al., 1994; Wu et al., 2003; Yang et al., 2010). Future studies will determine the effects of Wnt5a-induced PKC dephosphorylation on RGC protection, neurite growth and axonal regrowth.

In conclusion, we demonstrated that Wnt5a significantly improves RGC survival and optic nerve regeneration in an adult ONC mouse model. Furthermore, Wnt5a induces multiple prosurvival and proregenerative pathways in RGCs and other cell types in the retina. Future studies using pharmacologic inhibitors or RGC-specific genetic deletions of the identified downstream genes will determine whether Wnt5a-induced CamKII/CREB and JNK/STAT3 signaling and downregulation of PKC or PTEN act in parallel or synergistic pathways to induce RGC survival and axonal growth. Finally, further studies using sustained induction of Wnt5a signaling will provide information on long-term effects of Wnt5a on RGC survival and optic nerve regeneration and will determine whether Wnt5a would be sufficient to induce optic nerve axons to reach appropriate brain targets.

## Synthesis

Reviewing Editor: Matthew Grubb, King’s College London Decisions are customarily a result of the Reviewing Editor and the peer reviewers coming together and discussing their recommendations until a consensus is reached. When revisions are invited, a fact-based synthesis statement explaining their decision and outlining what is needed to prepare a revision will be listed below. The following reviewer(s) agreed to reveal their identity: NONE.

Reviewer 1:

The manuscript “Identification of a novel axon regeneration role for non-canonical Wnt signaling in the adult retina after injury” describes Wnt5a has effects in promoting optic nerve regeneration and protecting RGC survival from nerve injury, as well as the possible downstream signaling pathways. There are some concerns with the major design/technical issues. Here are the main points that authors need to address or consider.

1. Authors presented the normal Wnt5a is high in retinal ganglion cells, as well as other cells in retina, and the level is even higher after optic nerve injury. However, authors did not explain why this high level of endogenous Wnt5a does not induce axon regeneration and why the pro-regenerative Wnt5a has to be exogenous, and what’s the difference.

2. In the in vitro RGC cultures, RGCs are at postnatal P10-12 which are in the development and can not represent the status of adult RGCs. Therefore, the effects in these cultures are not meaningful to link to the axon regeneration in adult animals.

3. In the evaluation of optic nerve regeneration, authors did not describe how to avoid repeated counting of the same axons within a distance range (for example, 200-500 μm, etc.): axons do not extend straightly. In one nerve section, the axons might appear at 250 μm, and appear again at 400 μm. How did authors avoid counting them twice? It is well recognized in the field that, the axon quantitation is at a certain distance (like 200 μm, or 500 μm, etc.), but not a distance range (like from 200 to 500 μm). In addition, the length of one single longest axon does not make sense to represent axon regeneration. How do you know the sections contain the real longest axon in that nerve?

4. RGC survival: please use whole-mount retina staining and counting to quantify RGC survival, not using the retina cross sections, since the RGCs distribution is not even in the retina and the cross sections are not representative.

5. For Western blots and qPCRs: the whole retina samples do not represent the signaling in RGCs, especially the RGC numbers are only a small percentage in the whole retina. So the data are not meaningful.

6. There are no detailed statistical tests described for each quantitation.

Reviewer 2:

In this paper titled “Identification of a novel axon regeneration role for non-canonical Wnt signaling in the adult retina after injury” the authors examine the effects of Wnt5a on neuroprotection and axon regeneration after optic nerve injury. Although expression and functions of molecules involved in Wnt signaling pathways are well documented during development, its potential function in axon regeneration after optic nerve injury has not been well studied. Authors demonstrate in this paper that injections of exogenous Wnt5a enhance RGC survival and axon regeneration after injury and modulate regenerative and neuroprotective downstream signals. These findings are important in the field. However, there are two major issues that need to be addressed before the manuscript is suitable for publication.

Major Comments:

1. In the results section the final sentence of the second paragraph reads “Therefore, ONC is associated with Wnt5a upregulation (Fig 2), suggesting that Wnt5a upregulation may play an important role in the response of the retina after ONC injury”. Though the results do show functions of exogenous Wnt5a after injury, more evidence needs to be provided to suggest that endogenous Wnt5a is actually playing an important role. One way to do this is that perform Wnt5a knockdown experiments to see how RGC survival is affected after ONC since ∼20% RGCs still survive two weeks after ONC. If Wnt5a does play an important role, a decrease in RGC survival would be expected. Wnt5a expression was only examined at one timepoint (3 days after ONC). It would be most beneficial to see how Wnt5a expression changes over time. If Wnt5a expression falls over time as RGC death increases, then this could also provide more evidence in its important role in the injured retina.

2. Figures 6-11 only explore the downstream signals of Wnt5a in the uninjured retina and only at one timepoint (24hours after injection). Though these experiments do indeed document the effects of Wnt5a under normal conditions, it does not sufficiently explore these pathways in the injured retina. It would be most advantageous to the authors if they checked expression and phosphorylation of these proteins in the injured retina with Wnt5a injections. This could also be coupled with additional timepoints after injury to show how long the effects of Wnt5a injections last.

Minor Comments:

1. Neurite complexity is not defined. Is that the number of branch points on a neurite or something else? If the former is correct, consider saying that explicitly or at the very least mention it in the results. In addition, based on these findings, it would be great to discuss whether Wnt5a could promote “axon sprouting” and/or “axon regeneration”.

2. Second paragraph of the introduction the first parentheses in “(RAC1)” is grey not black.

## Author Response

June 23, 2022

Editor, eNeuro

Dear Dr. Grubb,

Thank you for forwarding us the reviewer comments on our manuscript “Identification of a novel axon regeneration role for non-canonical Wnt signaling in the adult retina after injury,”.

We appreciate the reviewers’ evaluations of our manuscript and thank them for their constructive criticism and for the positive comments on our work. The responses to all comments are described point-by-point in the letter below and are indicated in the text in red font. We hope that the incorporation of these suggestions has improved the manuscript and that it is now acceptable for publication.

Thank you very much for your consideration.

Editor’s Comments to Author:

Reviewers’ Comments to Author:

Reviewer: 1

---------------------------------------------

Synthesis of Reviews:

Both Reviewers stress the potential interest and importance of this work, but underline the need for substantial revisions before the conclusions in the manuscript are fully supported by the data.

In particular, the roles of endogenous versus exogenous Wnt5a must be addressed as suggested by Reviewer 2. New experiments are required to describe the time course of Wnt5a expression after injury, and the effect of endogenous Wnt5a loss-of-function on RGC regeneration.

For relevance to regeneration, factors acting downstream of Wnt5a also need to be described in the *injured* retina, ideally (though not necessarily) at multiple post-injury time points as suggested by Reviewer 2.

Both reviewers also identify areas where quantification and statistical analysis must either be re-done using more appropriate approaches, or at the very least strongly justified with additional controls and/or arguments.

The relevance of whole-retina analyses and cell culture assays for the central claims of the manuscript should also be directly addressed (see Reviewer 1).

Reviewer 1:

The manuscript “Identification of a novel axon regeneration role for non-canonical Wnt signaling in the adult retina after injury” describes Wnt5a has effects in promoting optic nerve regeneration and protecting RGC survival from nerve injury, as well as the possible downstream signaling pathways. There are some concerns with the major design/technical issues. Here are the main points that authors need to address or consider.

1. Authors presented the normal Wnt5a is high in retinal ganglion cells, as well as other cells in retina, and the level is even higher after optic nerve injury. However, authors did not explain why this high level of endogenous Wnt5a does not induce axon regeneration and why the pro-regenerative Wnt5a has to be exogenous, and what’s the difference.

Thank you for this comment. We speculate that endogenous Wnt5a is elevated as an intrinsic protective response to tissue damage. However, in the presence of significant or continuing damage, increased endogenous Wnt5a and activation of its signaling pathways are not sufficient to prevent degeneration. In contrast, exogenous Wnt5a would be predicted to increase these protective pathways further, which would elevate the threshold for apoptosis and reduce RGC degeneration. The current manuscript is focused on therapeutic potential of exogenous Wnt5a and all our analyses used Wnt5a delivery to test the effect on regeneration and neuronal protection.

Although several studies in addition to ours have identified anti-apoptotic effects of exogenous Wnt5a (Zhou, Chen et al. 2017)(Masckauchan, Agalliu et al. 2006, Blakely, Bye et al. 2011), an alternative possibility is that elevated endogenous Wnt5a in the retina may reflect changes unrelated to neuroprotection or regeneration, such as tissue remodeling or inflammation, and that only exogenous Wnt5a is neuroprotective. Therefore, future studies using Wnt5a knockout mice will be required to fully characterize the role of endogenous Wnt5a after injury.

2. In the in vitro RGC cultures, RGCs are at postnatal P10-12 which are in the development and can not represent the status of adult RGCs. Therefore, the effects in these cultures are not meaningful to link to the axon regeneration in adult animals.

The reviewer raises an important point. Unfortunately, it is not possible to culture viable RGCs from adult mice and the majority of publications using cultured RGCs obtain them from young post-natal or embryonic animals. We used RGCs from animals at the oldest time point that they can be reliably cultured, which is an advance over studies that use embryonic RGCs. Also, the axons of these RGCs are severed by removing them from the eye and neurite/axonal regrowth can be considered as regeneration after an injury. We agree with the reviewer that they are not the same as adult RGCs but do believe that our findings in these neurons are meaningful because RGC development is fully complete by birth and these postnatal RGCs do not represent developing neurons. We have added a sentence to the Discussion (2nd paragraph) that states that an important caveat is that technical limitations preclude our ability to culture adult RGCs and we instead used RGCs from young post-natal animals.

3. In the evaluation of optic nerve regeneration, authors did not describe how to avoid repeated counting of the same axons within a distance range (for example, 200-500 μm, etc.): axons do not extend straightly. In one nerve section, the axons might appear at 250 μm, and appear again at 400 μm. How did authors avoid counting them twice? It is well recognized in the field that, the axon quantitation is at a certain distance (like 200 μm, or 500 μm, etc.), but not a distance range (like from 200 to 500 μm).

Thank you for this suggestion. We have replaced the axon quantification range data with the specific distance data as suggested. The new data is in revised Figure 5B, which replaces previous figure 5C. These results show significantly more axons in the 50 ng Wnt5a treated group at all sites (assessed at 200 μm intervals from 400-1400 μm) compared to saline (Fig 5B).

In addition, the length of one single longest axon does not make sense to represent axon regeneration. How do you know the sections contain the real longest axon in that nerve?

Thank you for this comment. We actually quantified axon regeneration using axon counts at specific intervals (see revised Fig 5B). The single longest axon per nerve data in Fig 5C was intended not to quantify axon regeneration but to indicate the potential for regrowth of axons after Wnt5a injection compared to control treatment. The data in Fig 5C shows that four of the animals in the 50ng Wnt5a group had axons >2000 μm, and one animal had an axon up to 2700 μm, which is further evidence of the regeneration promoting effect of Wnt5a. In contrast, only one animal in the saline injected axons had axons as long as 1000 μm and the majority of animals were up to 800 μm. Therefore, Fig 5C was included to show the robust axonal regrowth that we observed in multiple animals injected with Wnt5a.

Although it is possible that the real longest axon was missed, we believe that our procedure for axon counting most likely identified the longest axon in the nerve for the following reasons: 1) Regrown axons are labeled by CTB and only CTB-positive axons were measured, 2) the entire nerve was sectioned longitudinally, 3) all of the sections from the nerve were examined for axonal growth, and 4) the longest axon detected in all the sections for a given nerve for each animal is measured. We then calculated the average length of the longest axon across all animals for each experimental treatment. Therefore, although it is plausible that we missed longer axons, we do not believe that would be the case given our procedure that captures all CTB-labeled axons. We use CTB instead of a microtubule marker to label the axons to confirm that they are regenerated and not delayed atrophy. However, it is possible that a longer axon may have been missed if it was close to the edge of the nerve; we do not measure these “edge axons” because it is difficult to distinguish them from the non-specific fluorescence at the edge of the nerve. We have added further explanation of these results to the manuscript (Results, pg 9) and relabeled the longest axon figure 5C for clarity.

4. RGC survival: please use whole-mount retina staining and counting to quantify RGC survival, not using the retina cross sections, since the RGCs distribution is not even in the retina and the cross sections are not representative.

Thank you for the opportunity to explain our approach. We use cross-sections obtained from the optic nerve region of all mice to make sure that equivalent regions of the retina are tested in each animal in every group. It is important to note that several studies indicate that there is homogenous loss of RGCs after ONC and axotomy (Mead et al., 2014), in contrast to sectorial RGC loss in glaucoma models (Soto et al., 2011). Importantly, Mead et al. compared retinal cross sections to wholemounts for quantifying RGC death after ONC and concluded that “90% RGC death 3 weeks after ONC in rats can be reported with equal fidelity in either wholemounts or radial retinal sections” (Mead et al., 2014). Furthermore, Mead concluded that sampling around the optic nerve head in sections, which we did, is a reliable estimate of RGC loss in conditions with homogenous loss of RGCs, such as after ONC. This is in contrast to glaucoma models where Mead et al state that counting in regions at multiple distances from the optic nerve head should be performed.

Furthermore, a recent paper by Kingston et al. examined differential vulnerability of RGCs after optic nerve injury in the mouse and showed no regional variation of RGC death and even distribution of surviving RGCs across the retina when measured at 2 weeks post-crush, despite the initial regional differential at 5 days post-crush (Fig 3 in Kingston et al., 2021). We also used the 2 week timepoint, which makes regional variation of RGC loss less likely in our study.

Additionally, an advantage of cross-sections is that they allow identification of displaced RGCs that are in the IPL, which degenerate at the same rate as RGCs within the GCL (Nadal-Nicolás et al., 2014). It is difficult to image these displaced RGCs in flatmounts, which would lead to undercounting. Furthermore, both cross-sections and flat mounts are commonly used in the field because both reliable measurements of RGC survival after injury. In summary, we feel that the cross-sections are an appropriate analysis due to the combination of lack of regional variation in the ONC model at this timepoint, analysis of the same retinal area in all animals, and specific limitations of flatmounts that may make undercounting a possibility.

5. For Western blots and qPCRs: the whole retina samples do not represent the signaling in RGCs, especially the RGC numbers are only a small percentage in the whole retina. So the data are not meaningful.

The reviewer raises an important point. Approximately 70% of the cells in the mouse retina are photoreceptors while RGCs are only 5%. However, photoreceptors are not responding to optic nerve crush or Wnt5a injection based on our immunohistochemistry experiments in Figures 7-10. Therefore, while the Western blot data reflects changes in the retina as a whole, and most likely the combined responses of RGCs and Muller glia, the Western blot data is consistent with immunohistochemistry data that localized the signaling proteins in the RGCs. Therefore, we feel that the data is meaningful and provides insight into potential pathways that are stimulated or suppressed by Wnt5a. We added a sentence to the Results (pg 10, 2nd paragraph) discussing this point.

6. There are no detailed statistical tests described for each quantitation.

Our apologies for this omission. We have added the statistical tests to the Methods section (pg 7) and a detailed document with all the statistical results is included as a supplemental file.

Reviewer 2:

In this paper titled “Identification of a novel axon regeneration role for non-canonical Wnt signaling in the adult retina after injury” the authors examine the effects of Wnt5a on neuroprotection and axon regeneration after optic nerve injury. Although expression and functions of molecules involved in Wnt signaling pathways are well documented during development, its potential function in axon regeneration after optic nerve injury has not been well studied. Authors demonstrate in this paper that injections of exogenous Wnt5a enhance RGC survival and axon regeneration after injury and modulate regenerative and neuroprotective downstream signals. These findings are important in the field. However, there are two major issues that need to be addressed before the manuscript is suitable for publication.

Major Comments:

1a. In the results section the final sentence of the second paragraph reads “Therefore, ONC is associated with Wnt5a upregulation (Fig 2), suggesting that Wnt5a upregulation may play an important role in the response of the retina after ONC injury”. Though the results do show functions of exogenous Wnt5a after injury, more evidence needs to be provided to suggest that endogenous Wnt5a is actually playing an important role. One way to do this is that perform Wnt5a knockdown experiments to see how RGC survival is affected after ONC since ∼20% RGCs still survive two weeks after ONC. If Wnt5a does play an important role, a decrease in RGC survival would be expected.

The reviewer raises an important point and is correct that we currently have no evidence that endogenous Wnt5a is playing an important role after injury, despite determining that that exogenous Wnt5a is protective. Therefore, we have removed the sentence about the role of endogenous Wnt5a quoted by the reviewer (pg 8).

Additionally, we agree that Wnt5a knockdown experiments would be informative. However, given that such knock-down would require extensive experimentation and optimization, and the main objective of the paper is identifying whether exogenous Wnt5a induces neuroprotection and axonal regeneration after injury, we feel that the suggested knockdown experiments would be better performed in a future study. We have added a sentence to the Discussion to that effect (pg 12, 2nd paragraph).

1b. Wnt5a expression was only examined at one timepoint (3 days after ONC). It would be most beneficial to see how Wnt5a expression changes over time. If Wnt5a expression falls over time as RGC death increases, then this could also provide more evidence in its important role in the injured retina.

We agree that investigating the expression of endogenous Wnt5a over time would be interesting and performing the time course that the reviewer suggested would be a good approach to investigate endogenous Wnt5a. However, investigating endogenous Wnt5a is beyond the scope of the current manuscript that focuses on potential therapeutic effects of exogenous Wnt5a. Therefore, similar to the response to question 1, we feel that analysis of endogenous Wnt5a should be the focus of a future study.

2. Figures 6-11 only explore the downstream signals of Wnt5a in the uninjured retina and only at one timepoint (24hours after injection). Though these experiments do indeed document the effects of Wnt5a under normal conditions, it does not sufficiently explore these pathways in the injured retina. It would be most advantageous to the authors if they checked expression and phosphorylation of these proteins in the injured retina with Wnt5a injections. This could also be coupled with additional timepoints after injury to show how long the effects of Wnt5a injections last.

The reviewer raises an important point and one that we had carefully considered during the design of the experiments. We chose to inject Wnt5a into uninjured naïve retinas instead of injured retinas in order to determine activation of Wnt5a pathways without the complication of simultaneous injury tissue responses. In other words, Wnt5a signaling in the naïve retinas in Figs 6-11 shows the potential pathways that could be stimulated by Wnt5a, whereas in injured retinas these pathways would likely also be stimulated as part of a glial response in addition to Wnt5a. As an example, the JNK/Stat3 pathway is induced by injury and contributes to the inflammatory and glial response, and if we had tested expression in injured retinas we would not have been able to determine whether Wnt5a also induces JNK/Stat3. Therefore, the experimental design we used identified potential pathways that can be stimulated by Wnt5a. Confirmation that these pathways are associated with the regenerative and neuroprotective effects of Wnt5a will require examination of the pathways in injury over time, as the reviewer suggests, as well as meticulous inhibitor and agonist studies. Such analysis is planned as part of a future study. We have added a sentence to the Discussion (pg 12, 13) that summarizes the limitation raised by the reviewer here. We also modified multiple sentences in the Results (pg 9 & 10) and Discussion (pg 10), and restated our conclusions, to make it clear that the signaling pathways were analyzed in uninjured retinas and that the contribution of these pathways to Wnt5a-mediated neuroprotection or regeneration requires further study.

Minor Comments:

1. Neurite complexity is not defined. Is that the number of branch points on a neurite or something else? If the former is correct, consider saying that explicitly or at the very least mention it in the results. In addition, based on these findings, it would be great to discuss whether Wnt5a could promote “axon sprouting” and/or “axon regeneration”.

We defined neurite complexity as the number of branch points on the neurites, following the approach of Pino et al. 2014 and Rosso et al. 2005. We have added the definition to the Methods and Results sections to clarify what was measured.

Regarding axon sprouting, Geoffroy and Zheng (2014) define sprouting is any new axonal growth from uninjured neurons whereas regeneration is axon growth from injured neurons. Regeneration can occur from the cut end or tip of injured axons, from the shaft of injured axons, or from extending pre-existing uninjured axons. Our analysis tested injured RGCs in vivo and in vitro: the RGC axons in vivo were injured by crush and the axons in the cultured RGCs were injured by axon transection and removal from the eye. Therefore, our data demonstrated RGC axonal regeneration from Wnt5a. We do not know at this point whether Wnt5a can promote axonal sprouting on RGCs and it remains an important topic of future work.

2. Second paragraph of the introduction the first parentheses in “(RAC1)” is grey not black.

Thank you for pointing out this error, the font has been changed to black.

References

Geoffroy and Zheng. (2014) Myelin-associated inhibitors in axonal growth after CNS injury Current Opinion in Neurobiology, 27:31-38

Kingston et al. (2021) Serotonin transporter-mediated molecular axis regulates regional retinal ganglion cell vulnerability and axon regeneration after nerve injury. PLoS Genet.

17(11):e1009885.

Masckauchán et al. (2006) Wnt5a signaling induces proliferation and survival of endothelial cells in vitro and expression of MMP-1 and Tie-2. Mol Biol Cell. 17(12):5163-72.

Mead et al. (2014) Comparative evaluation of methods for estimating retinal ganglion cell loss in retinal sections and wholemounts. PLoS One.9(10):e110612..

Nadal-Nicolás et al. (2014) Displaced retinal ganglion cells in albino and pigmented rats. Front Neuroanat. 8:99.

Pino D et al. (2011) Wnt5a controls neurite development in olfactory bulb interneurons. ASN Neuro. 3(3):e00059.

Rosso et al. (2005) Wnt signaling through Dishevelled, Rac and JNK regulates dendritic development. Nat Neurosci. 8:34-42.

Soto et al. (2011) Retinal ganglion cell loss in a rat ocular hypertension model is sectorial and involves early optic nerve axon loss. Invest Ophthalmol Vis Sci 52(1):434-41.

Zhou et al. (2017). Wnt5a Promotes Cortical Neuron Survival by Inhibiting Cell-Cycle Activation. Front Cell Neurosci 11: 281.
