# Supplementary material for: Identification of a Novel Axon Regeneration Role for Noncanonical Wnt Signaling in the Adult Retina after Injury
Source: eNeuro. 2022 Aug 10;9(4):ENEURO.0182-22.2022. doi: 10.1523/ENEURO.0182-22.2022 (PMC9373906; doi:10.1523/ENEURO.0182-22.2022)
Supplement: Extended Data 1 — Statistics information Identification of a Novel Axon Regeneration Role for Non-Canonical Wnt Signaling in the Adult Retina After Injury. Download Extended Data 1, DOCX file. [file enu-eN-NWR-0182-22-s02.docx]

**Statistics information**

**Identification of a Novel Axon Regeneration Role for Non-Canonical Wnt Signaling in the Adult Retina After Injury**

**Fig 2. QPCR quantification of Wnt5a gene expression 3 days after ONC injury showed a significant upregulation in ONC injured retinas compared to uninjured retinas.**

Column B ONC injury

vs. vs.

Column A Un Injured

**Unpaired t test**

P value 0.0104

P value summary *

Significantly different (P < 0.05)? Yes

One- or two-tailed P value? Two-tailed

t, df t=4.555 df=4

How big is the difference?

Mean ± SEM of column A 1.007 ± 0.07513, n=3

Mean ± SEM of column B 4.553 ± 0.7751, n=3

Difference between means 3.547 ± 0.7787

95% confidence interval 1.385 to 5.709

R squared (eta squared) 0.8384

F test to compare variances

F, DFn, Dfd 106.4, 2, 2

P value 0.0186

P value summary *

Significantly different (P < 0.05)? Yes

**Fig 3: Wnt5a treatment induced neurite growth, neurite complexity and neurite number in RGC primary cultures.**

**E) average neurite length**

ANOVA summary

F 261.1

P value <0.0001

P value summary ****

Significant diff. among means (P < 0.05)? Yes

R square 0.9899

Brown-Forsythe test

F (DFn, DFd) 0.9902 (3, 8)

P value 0.445

P value summary ns

Are SDs significantly different (P < 0.05)? No

ANOVA table SS DF MS F (DFn, DFd) P value

Treatment (between columns) 226093 3 75364 F (3, 8) = 261.1 P<0.0001

Residual (within columns) 2309 8 288.6

Total 228402 11

**Tukey's multiple comparisons test**

Mean Diff. 95.00% CI of diff. Significant? Summary Adjusted P Value

BSA treated vs. 25ng Wnt5A -102.8 -147.2 to -58.38 Yes *** 0.0003 A-B

BSA treated vs. 50ng Wnt5a -201.3 -245.8 to -156.9 Yes **** <0.0001 A-C

BSA treated vs. 100ng Wnt5a -372.4 -416.8 to -328 Yes **** <0.0001 A-D

25ng Wnt5A vs. 50ng Wnt5a -98.55 -143 to -54.13 Yes *** 0.0005 B-C

25ng Wnt5A vs. 100ng Wnt5a -269.6 -314 to -225.2 Yes **** <0.0001 B-D

50ng Wnt5a vs. 100ng Wnt5a -171.1 -215.5 to -126.6 Yes **** <0.0001 C-D

**Fig.3 E) Average neurite number**

Table Analyzed One-way ANOVA data

Data sets analyzed A : BSA treated B : 25ng Wnt5A C : 50ng Wnt5a D : 100ng Wnt5a

ANOVA summary

F 17.17

P value 0.0008

P value summary ***

Significant diff. among means (P < 0.05)? Yes

R square 0.8656

Brown-Forsythe test

F (DFn, DFd) 0.3884 (3, 8)

P value 0.7646

P value summary ns

Are SDs significantly different (P < 0.05)? No

Tukey's multiple comparisons test

Mean Diff. 95.00% CI of diff. Significant? Summary Adjusted P Value

BSA treated vs. 25ng Wnt5A -1.56 -2.674 to -0.4459 Yes ** 0.0088 A-B

BSA treated vs. 50ng Wnt5a -2.433 -3.547 to -1.319 Yes *** 0.0005 A-C

BSA treated vs. 100ng Wnt5a -1.653 -2.767 to -0.5393 Yes ** 0.0063 A-D

25ng Wnt5A vs. 50ng Wnt5a -0.8733 -1.987 to 0.2407 No ns 0.1325 B-C

25ng Wnt5A vs. 100ng Wnt5a -0.09333 -1.207 to 1.021 No ns 0.9927 B-D

50ng Wnt5a vs. 100ng Wnt5a 0.78 -0.3341 to 1.894 No ns 0.1916 C-D

**Fig.3 E) Average neurite complexity (branch site number)**

Table Analyzed One-way ANOVA data

Data sets analyzed A : BSA treated B : 25ng Wnt5A C : 50ng Wnt5a D : 100ng Wnt5a

ANOVA summary

F 16.7

P value 0.0008

P value summary ***

Significant diff. among means (P < 0.05)? Yes

R square 0.8623

Brown-Forsythe test

F (DFn, DFd) 0.5435 (3, 8)

P value 0.6661

P value summary ns

Are SDs significantly different (P < 0.05)? No

Bartlett's test

Bartlett's statistic (corrected)

P value

P value summary

Are SDs significantly different (P < 0.05)?

ANOVA table SS DF MS F (DFn, DFd) P value

Treatment (between columns) 5.659 3 1.886 F (3, 8) = 16.7 P=0.0008

Tukey's multiple comparisons test

Mean Diff. 95.00% CI of diff. Significant? Summary Adjusted P Value

BSA treated vs. 25ng Wnt5A -1.587 -2.466 to -0.7078 Yes ** 0.0019 A-B

BSA treated vs. 50ng Wnt5a -1.763 -2.642 to -0.8845 Yes *** 0.0009 A-C

BSA treated vs. 100ng Wnt5a -1.135 -2.014 to -0.2558 Yes * 0.0139 A-D

25ng Wnt5A vs. 50ng Wnt5a -0.1767 -1.056 to 0.7022 No ns 0.9148 B-C

25ng Wnt5A vs. 100ng Wnt5a 0.452 -0.4269 to 1.331 No ns 0.4075 B-D

50ng Wnt5a vs. 100ng Wnt5a 0.6287 -0.2502 to 1.508 No ns 0.1794 C-D

**Fig 4B.** **Quantification of RBPMS immunopositive cells that co-localized with DAPI showing significantly increased RGC density in 20 ng and 50 ng Wnt5a injected retinas compared to saline injected retinas.**

Table Analyzed One-way ANOVA data

Data sets analyzed A : Saline B : Wnt5a 20ng C : Wnt5a 50ng

ANOVA summary

F 18.54

P value 0.0027

P value summary **

Significant diff. among means (P < 0.05)? Yes

R square 0.8607

Brown-Forsythe test

F (DFn, DFd) 0.3008 (2, 6)

P value 0.7507

P value summary ns

Are SDs significantly different (P < 0.05)? No

Bartlett's test

Bartlett's statistic (corrected)

P value

P value summary

Are SDs significantly different (P < 0.05)?

ANOVA table SS DF MS F (DFn, DFd) P value

Treatment (between columns) 1249 2 624.6 F (2, 6) = 18.54 P=0.0027

Residual (within columns) 202.1 6 33.69

Total 1451 8

Tukey's multiple comparisons test

Mean Diff. 95.00% CI of diff. Significant? Summary Adjusted P Value

Saline vs. Wnt5a 20ng -19.85 -34.39 to -5.314 Yes * 0.0136 A-B

Saline vs. Wnt5a 50ng -28.06 -42.6 to -13.52 Yes ** 0.0025 A-C

Wnt5a 20ng vs. Wnt5a 50ng -8.21 -22.75 to 6.33 No ns 0.2692 B-C

**Fig 5BC Axonal regeneration 2 weeks after ONC injury following a single intravitreal injection of Wnt5a.**

**5C) Average longest axon**

Table Analyzed longest axon sal, 20, 50

Data sets analyzed A : Saline B : 20 ng Wnt5a C : 50 ng Wnt5a

ANOVA summary

F 23.44

P value <0.0001

P value summary ****

Significant diff. among means (P < 0.05)? Yes

R square 0.7576

Brown-Forsythe test

F (DFn, DFd) 2.02 (2, 15)

P value 0.1672

P value summary ns

Are SDs significantly different (P < 0.05)? No

Bartlett's test

Bartlett's statistic (corrected) 4.147

P value 0.1258

P value summary ns

Are SDs significantly different (P < 0.05)? No

Tukey's multiple comparisons test

Mean Diff. 95.00% CI of diff. Significant? Summary Adjusted P Value

Saline vs. 20 ng Wnt5a -137.9 -675.3 to 399.5 No ns 0.7861 A-B

Saline vs. 50 ng Wnt5a -1284 -1840 to -728.7 Yes **** <0.0001 A-C

20 ng Wnt5a vs. 50 ng Wnt5a -1147 -1657 to -636 Yes **** <0.0001 B-C

**5B) Average axon count**

Data sets analyzed A : Saline 400 B : 20ng Wnt5a 400 C : 50ng Wnt5a 400

ANOVA summary

F 8.196

P value 0.0039

P value summary **

Significant diff. among means (P < 0.05)? Yes

R square 0.5222

Brown-Forsythe test

F (DFn, DFd) 0.144 (2, 15)

P value 0.8671

P value summary ns

Are SDs significantly different (P < 0.05)? No

Bartlett's test

Bartlett's statistic (corrected) 0.08781

P value 0.9570

P value summary ns

Are SDs significantly different (P < 0.05)? No

ANOVA table SS DF MS F (DFn, DFd) P value

Treatment (between columns) 4657 2 2329 F (2, 15) = 8.196 P=0.0039

Residual (within columns) 4262 15 284.1

Total 8919 17

Tukey's multiple comparisons test

Mean Diff. 95.00% CI of diff. Significant? Summary Adjusted P Value

Saline 400 vs. 20ng Wnt5a 400 6.8 -18.84 to 32.44 No ns 0.7734 A-B

Saline 400 vs. 50ng Wnt5a 400 -29.66 -56.17 to -3.146 Yes * 0.0276 A-C

20ng Wnt5a 400 vs. 50ng Wnt5a 400 -36.46 -60.82 to -12.1 Yes ** 0.0039 B-C

Data sets analyzed D : Saline 600 E : 20ng Wnt5a 600 F : 50ng Wnt5a 600

ANOVA summary

F 9.421

P value 0.0018

P value summary **

Significant diff. among means (P < 0.05)? Yes

R square 0.5257

Brown-Forsythe test

F (DFn, DFd) 1.223 (2, 17)

P value 0.3189

P value summary ns

Are SDs significantly different (P < 0.05)? No

Bartlett's test

Bartlett's statistic (corrected) 2.937

P value 0.2302

P value summary ns

Are SDs significantly different (P < 0.05)? No

ANOVA table SS DF MS F (DFn, DFd) P value

Treatment (between columns) 1321 2 660.5 F (2, 17) = 9.421 P=0.0018

Residual (within columns) 1192 17 70.11

Total 2513 19

Data summary

Number of treatments (columns) 3

Number of values (total) 20

Number of families 1

Number of comparisons per family 3

Alpha 0.05

Tukey's multiple comparisons test Mean Diff. 95.00% CI of diff. Significant? Summary Adjusted P Value

Saline 600 vs. 20ng Wnt5a 600 5.283 -6.199 to 16.76 No ns 0.4803 D-E

Saline 600 vs. 50ng Wnt5a 600 -14.43 -26.38 to -2.475 Yes * 0.0171 D-F

20ng Wnt5a 600 vs. 50ng Wnt5a 600 -19.71 -31.66 to -7.758 Yes ** 0.0015 E-F

Test details Mean 1 Mean 2 Mean Diff. SE of diff. n1 n2 q DF

Saline 600 vs. 20ng Wnt5a 600 9.95 4.667 5.283 4.476 7 7 1.669 17

Saline 600 vs. 50ng Wnt5a 600 9.95 24.38 -14.43 4.658 7 6 4.379 17

20ng Wnt5a 600 vs. 50ng Wnt5a 600 4.667 24.38 -19.71 4.658 7 6 5.983 17

Data sets analyzed D : Saline 800 E : 20ng Wnt5a 800 F : 50ng Wnt5a 800

ANOVA summary

F 11.96

P value 0.0008

P value summary ***

Significant diff. among means (P < 0.05)? Yes

R square 0.6145

Brown-Forsythe test

F (DFn, DFd) 5.651 (2, 15)

P value 0.0148

P value summary *

Are SDs significantly different (P < 0.05)? Yes

Bartlett's test

Bartlett's statistic (corrected) 5.053

P value 0.0799

P value summary ns

Are SDs significantly different (P < 0.05)? No

ANOVA table SS DF MS F (DFn, DFd) P value

Treatment (between columns) 664.9 2 332.5 F (2, 15) = 11.96 P=0.0008

Residual (within columns) 417.1 15 27.81

Total 1082 17

Tukey's multiple comparisons test

Mean Diff. 95.00% CI of diff. Significant? Summary Adjusted P Value

Saline 800 vs. 20ng Wnt5a 800 0.6262 -7.394 to 8.646 No ns 0.9776 D-E

Saline 800 vs. 50ng Wnt5a 800 -12.52 -20.81 to -4.223 Yes ** 0.0037 D-F

20ng Wnt5a 800 vs. 50ng Wnt5a 800 -13.14 -20.76 to -5.523 Yes ** 0.0012 E-F

Data sets analyzed G : Saline 1000 H : 20ng Wnt5a 1000 I : 50ng Wnt5a 1000

ANOVA summary

F 21.69

P value <0.0001

P value summary ****

Significant diff. among means (P < 0.05)? Yes

R square 0.7431

Brown-Forsythe test

F (DFn, DFd) 19.17 (2, 15)

P value <0.0001

P value summary ****

Are SDs significantly different (P < 0.05)? Yes

Bartlett's test

Bartlett's statistic (corrected) 27.07

P value <0.0001

P value summary ****

Are SDs significantly different (P < 0.05)? Yes

ANOVA table SS DF MS F (DFn, DFd) P value

Treatment (between columns) 131.3 2 65.65 F (2, 15) = 21.69 P<0.0001

Residual (within columns) 45.4 15 3.027

Total 176.7 17

Tukey's multiple comparisons test

Mean Diff. 95.00% CI of diff. Significant? Summary Adjusted P Value

Saline 1000 vs. 20ng Wnt5a 1000 0.007143 -2.639 to 2.653 No ns >0.9999 G-H

Saline 1000 vs. 50ng Wnt5a 1000 -5.725 -8.461 to -2.989 Yes *** 0.0002 G-I

20ng Wnt5a 1000 vs. 50ng Wnt5a 1000 -5.732 -8.246 to -3.218 Yes **** <0.0001 H-I

Data sets analyzed M : Saline 1200 N : 20ng Wnt5a 1200 O : 50ng Wnt5a 1200

ANOVA summary

F 3.652

P value 0.0551

P value summary ns

Significant diff. among means (P < 0.05)? No

R square 0.3597

Brown-Forsythe test

F (DFn, DFd) 3.458 (2, 13)

P value 0.0625

P value summary ns

Are SDs significantly different (P < 0.05)? No

Bartlett's test

Bartlett's statistic (corrected) +infinity

P value <0.0001

P value summary ****

Are SDs significantly different (P < 0.05)? Yes

ANOVA table SS DF MS F (DFn, DFd) P value

Treatment (between columns) 119.4 2 59.7 F (2, 13) = 3.652 P=0.0551

Residual (within columns) 212.5 13 16.35

Total 331.9 15

Data summary

Number of treatments (columns) 3

Number of values (total) 16

Number of families 1

Number of comparisons per family 3

Alpha 0.05

Tukey's multiple comparisons test Mean Diff. 95.00% CI of diff. Significant? Summary Adjusted P Value

Saline 1200 vs. 20ng Wnt5a 1200 -0.4 -7.152 to 6.352 No ns 0.9866 M-N

Saline 1200 vs. 50ng Wnt5a 1200 -5.833 -12.3 to 0.6314 No ns 0.0792 M-O

20ng Wnt5a 1200 vs. 50ng Wnt5a 1200 -5.433 -11.9 to 1.031 No ns 0.1050 N-O

Test details Mean 1 Mean 2 Mean Diff. SE of diff. n1 n2 q DF

Saline 1200 vs. 20ng Wnt5a 1200 0 0.4 -0.4 2.557 5 5 0.2212 13

Saline 1200 vs. 50ng Wnt5a 1200 0 5.833 -5.833 2.448 5 6 3.369 13

20ng Wnt5a 1200 vs. 50ng Wnt5a 1200 0.4 5.833 -5.433 2.448 5 6 3.138 13

Data sets analyzed J : Saline 1400 K : 20ng Wnt5a 1400 L : 50ng Wnt5a 1400

ANOVA summary

F 4.931

P value 0.0255

P value summary *

Significant diff. among means (P < 0.05)? Yes

R square 0.4314

Brown-Forsythe test

F (DFn, DFd) 4.577 (2, 13)

P value 0.0313

P value summary *

Are SDs significantly different (P < 0.05)? Yes

Bartlett's test

Bartlett's statistic (corrected) +infinity

P value <0.0001

P value summary ****

Are SDs significantly different (P < 0.05)? Yes

ANOVA table SS DF MS F (DFn, DFd) P value

Treatment (between columns) 22.46 2 11.23 F (2, 13) = 4.931 P=0.0255

Residual (within columns) 29.6 13 2.277

Total 52.06 15

Tukey's multiple comparisons test

Mean Diff. 95.00% CI of diff. Significant? Summary Adjusted P Value

Saline 1400 vs. 20ng Wnt5a 1400 -0.2 -2.72 to 2.32 No ns 0.9761 J-K

Saline 1400 vs. 50ng Wnt5a 1400 -2.542 -4.954 to -0.129 Yes * 0.0387 J-L

20ng Wnt5a 1400 vs. 50ng Wnt5a 1400 -2.342 -4.754 to 0.07102 No ns 0.0575 K-L

**Fig 6. (B) Normalized phospho protein band intensity with total protein band intensity of CamKII, JNK and PKC shows a significant upregulation of phospho CamKII and JNK in 20 ng Wnt5a injected retinas compared to saline injected retinas. In contrast, significant downregulation of phospho PKC was observed in 20 ng Wnt5a injected retinas compared to the saline injected retinas.**

**CamKII**

Column B 20ng Wnt5a

vs. vs.

Column A Saline

**Unpaired t test**

P value <0.0001

P value summary ****

Significantly different (P < 0.05)? Yes

One- or two-tailed P value? Two-tailed

t, df t=7.53 df=8

How big is the difference?

Mean ± SEM of column A 0.481 ± 0.09739, n=5

Mean ± SEM of column B 1.761 ± 0.1393, n=5

Difference between means 1.28 ± 0.17

95% confidence interval 0.8881 to 1.672

R squared (eta squared) 0.8764

F test to compare variances

F, DFn, Dfd 2.047, 4, 4

P value 0.5050

P value summary ns

Significantly different (P < 0.05)? No

**JNK**

Column B 20ng Wnt5a

vs. vs.

Column A Saline

**Unpaired t test**

P value 0.0007

P value summary ***

Significantly different (P < 0.05)? Yes

One- or two-tailed P value? Two-tailed

t, df t=5.285 df=8

How big is the difference?

Mean ± SEM of column A 0.4436 ± 0.04086, n=5

Mean ± SEM of column B 1.77 ± 0.2476, n=5

Difference between means 1.326 ± 0.2509

95% confidence interval 0.7475 to 1.905

R squared (eta squared) 0.7773

F test to compare variances

F, DFn, Dfd 36.72, 4, 4

P value 0.0041

P value summary **

Significantly different (P < 0.05)? Yes

**PKC**

Column B 20ng Wnt5a

vs. vs.

Column A Saline

**Unpaired t test**

P value 0.0002

P value summary ***

Significantly different (P < 0.05)? Yes

One- or two-tailed P value? Two-tailed

t, df t=6.571 df=8

How big is the difference?

Mean ± SEM of column A 2.103 ± 0.2807, n=5

Mean ± SEM of column B 0.2556 ± 0.01667, n=5

Difference between means -1.847 ± 0.2811

95% confidence interval -2.496 to -1.199

R squared (eta squared) 0.8437

F test to compare variances

F, DFn, Dfd 283.6, 4, 4

P value <0.0001

P value summary ****

Significantly different (P < 0.05)? Yes

**Fig 7 (B) Normalized phospho protein band intensity with total protein band intensity of CamKII, JNK, and** **PKC shows a significant upregulation of phospho CamKII, JNK and STAT3 in 50 ng Wnt5a injected retinas compared to saline injected retinas. In contrast, significant downregulation of phospho PKC was observed in 50 ng Wnt5a injected retinas compared to the saline injected retinas.**

**CamKII**

Column B 50ng Wnt5a

vs. vs.

Column A Saline

**Unpaired t test**

P value 0.0011

P value summary **

Significantly different (P < 0.05)? Yes

One- or two-tailed P value? Two-tailed

t, df t=5.002 df=8

How big is the difference?

Mean ± SEM of column A 0.641 ± 0.06917, n=5

Mean ± SEM of column B 1.859 ± 0.2335, n=5

Difference between means 1.218 ± 0.2435

95% confidence interval 0.6564 to 1.779

R squared (eta squared) 0.7577

F test to compare variances

F, DFn, Dfd 11.39, 4, 4

P value 0.0370

P value summary *

Significantly different (P < 0.05)? Yes

**JNK**

Column B 50ng Wnt5a

vs. vs.

Column A Saline

**Unpaired t test**

P value 0.0007

P value summary ***

Significantly different (P < 0.05)? Yes

One- or two-tailed P value? Two-tailed

t, df t=5.285 df=8

How big is the difference?

Mean ± SEM of column A 0.4436 ± 0.04086, n=5

Mean ± SEM of column B 1.77 ± 0.2476, n=5

Difference between means 1.326 ± 0.2509

95% confidence interval 0.7475 to 1.905

R squared (eta squared) 0.7773

F test to compare variances

F, DFn, Dfd 36.72, 4, 4

P value 0.0041

P value summary **

Significantly different (P < 0.05)? Yes

**PKC**

Column B 50ng Wnt5a

vs. vs.

Column A Saline

**Unpaired t test**

P value <0.0001

P value summary ****

Significantly different (P < 0.05)? Yes

One- or two-tailed P value? Two-tailed

t, df t=7.145 df=8

How big is the difference?

Mean ± SEM of column A 2.178 ± 0.2771, n=5

Mean ± SEM of column B 0.1911 ± 0.02232, n=5

Difference between means -1.986 ± 0.278

95% confidence interval -2.628 to -1.345

R squared (eta squared) 0.8645

F test to compare variances

F, DFn, Dfd 154.1, 4, 4

P value 0.0002

P value summary ***

Significantly different (P < 0.05)? Yes

**Fig 11: QPCR quantification of Stat3, Creb, Atf3, Cntf and Pten gene expression in retinas 1 day after intravitreal injections of 50 ng Wnt5a or saline injections (n=3). Bar diagrams shows a significant upregulation of Creb, Stat3, Atf3 and Cntf genes in Wnt5a injected retina compared to saline injected retinas. Significant downregulation of Pten gene expression was observed in Wnt5a injected retinas compared to saline injected retinas.**

**Stat3**

Column B 50ng Wnt5a

vs. vs.

Column A PBS injected

**Unpaired t test**

P value 0.0225

P value summary *

Significantly different (P < 0.05)? Yes

One- or two-tailed P value? Two-tailed

t, df t=3.613 df=4

How big is the difference?

Mean ± SEM of column A 1.077 ± 0.2864, n=3

Mean ± SEM of column B 2.55 ± 0.2902, n=3

Difference between means 1.473 ± 0.4077

95% confidence interval 0.3413 to 2.605

R squared (eta squared) 0.7655

F test to compare variances

F, DFn, Dfd 1.027, 2, 2

P value 0.9866

P value summary ns

Significantly different (P < 0.05)? No

**Creb**

Column B 50ng Wnt5a

vs. vs.

Column A PBS injected

**Unpaired t test**

P value 0.0028

P value summary **

Significantly different (P < 0.05)? Yes

One- or two-tailed P value? Two-tailed

t, df t=6.555 df=4

How big is the difference?

Mean ± SEM of column A 1.047 ± 0.2248, n=3

Mean ± SEM of column B 4.313 ± 0.4448, n=3

Difference between means 3.267 ± 0.4984

95% confidence interval 1.883 to 4.65

R squared (eta squared) 0.9148

F test to compare variances

F, DFn, Dfd 3.914, 2, 2

P value 0.4070

P value summary ns

Significantly different (P < 0.05)? No

**Atf3**

Column B 50ng Wnt5a

vs. vs.

Column A PBS injected

Unpaired t test

P value <0.0001

P value summary ****

Significantly different (P < 0.05)? Yes

One- or two-tailed P value? Two-tailed

t, df t=21.29 df=4

How big is the difference?

Mean ± SEM of column A 1.01 ± 0.1044, n=3

Mean ± SEM of column B 7.087 ± 0.2656, n=3

Difference between means 6.077 ± 0.2854

95% confidence interval 5.284 to 6.869

R squared (eta squared) 0.9913

F test to compare variances

F, DFn, Dfd 6.472, 2, 2

P value 0.2677

P value summary ns

Significantly different (P < 0.05)? No

**Cntf**

Column B 50ng Wnt5a

vs. vs.

Column A PBS injected

**Unpaired t test**

P value 0.0007

P value summary ***

Significantly different (P < 0.05)? Yes

One- or two-tailed P value? Two-tailed

t, df t=9.425 df=4

How big is the difference?

Mean ± SEM of column A 0.5967 ± 0.1141, n=3

Mean ± SEM of column B 2.82 ± 0.2065, n=3

Difference between means 2.223 ± 0.2359

95% confidence interval 1.568 to 2.878

R squared (eta squared) 0.9569

F test to compare variances

F, DFn, Dfd 3.277, 2, 2

P value 0.4677

P value summary ns

Significantly different (P < 0.05)? No

**Pten**

Column B 50ng Wnt5a

vs. vs.

Column A PBS injected

**Unpaired t test**

P value 0.0049

P value summary **

Significantly different (P < 0.05)? Yes

One- or two-tailed P value? Two-tailed

t, df t=5.632 df=4

How big is the difference?

Mean ± SEM of column A 1 ± 0.02517, n=3

Mean ± SEM of column B 0.67 ± 0.05292, n=3

Difference between means -0.33 ± 0.05859

95% confidence interval -0.4927 to -0.1673

R squared (eta squared) 0.888

F test to compare variances

F, DFn, Dfd 4.421, 2, 2

P value 0.3689

P value summary ns

Significantly different (P < 0.05)? No
